# An Ultra‐Selective and Humidity‐Resistant Room‐Temperature‐Operated NO_2_ Sensor Based on Black TiO_2_

**DOI:** 10.1002/advs.202509293

**Published:** 2025-08-11

**Authors:** Xuelan Cheng, Yizheng Liu, Wei Zhong, Shuai Li, Yan Li, Zixi Zhao, Chunlei Zhang, Jidong Shi, Hui Liu, Zonglong Zhu, Fang Xu

**Affiliations:** ^1^ Shenzhen Key Laboratory of Ultraintense Laser and Advanced Material Technology, Center for Advanced Material Diagnostic Technology, and College of Engineering Physics Shenzhen Technology University Shenzhen 518118 P. R. China; ^2^ Department of Chemistry City University of Hong Kong Kowloon Hong Kong 999077 P. R. China

**Keywords:** chemiresistive gas sensor, metal oxides, NO_2_, selectivity, TiO_2_

## Abstract

As a common gas‐sensing material, TiO_2_ is limited by poor gas selectivity and humidity immunity like many other metal oxides. Here, an ultra‐selective and humidity‐resistant room temperature‐operated NO_2_ sensor is developed using black TiO_2_ for the first time. Compared with conventional white TiO_2_, black TiO_2_ significantly enhances NO_2_ selectivity and humidity resistance by enhancing the NO_2_ response for ≥10 times while simultaneously suppressing responses to 7 common interfering gases and H_2_O adsorption. A wireless portable equipment is developed that exhibits the capabilities of identifying NO_2_ from a mixed atmosphere and recognizing environmental differences in practical scenarios. These improvements and different response types are attributed to the regulation of H_2_O adsorption: H_2_O layers are formed on white TiO_2_, leading to dominant ion‐proton conductivity and blocked gas–solid interactions, while H_2_O adsorption is suppressed and hydroxyl groups are formed on black TiO_2_, enhancing NO_2_ response. This study not only promotes a significant advancement and proves the feasibility of using common metal oxides for high‐performance NO_2_ detection at room temperature in a mixed environment irrespective of ambient humidity, but also offers valuable insights into the sensing mechanisms.

## Graphical abstract

A room‐temperature‐operated NO_2_ sensor that achieves ultrahigh selectivity and humidity resistance is developed using black TiO_2_ for the first time in this work. A portable sensing equipment capable of recognizing NO_2_ from a mixed atmosphere with several common interfering gases and identifying real environmental differences is developed correspondingly.

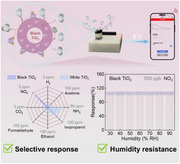

## 1 Introduction

Nitrogen dioxide (NO_2_) is a common air pollutant produced in large quantities from vehicle exhausts and industrial emissions. It causes environmental problems such as ground‐level ozone formation, acid rain, eutrophication, reduced visibility, and climate change. Prolonged exposure to NO_2_ causes chronic diseases, such as neurasthenic syndrome, respiratory tract inflammation, pulmonary fibrosis, and pulmonary edema.^[^ ^1^, ^2^, ^3^, ^4^ ^]^ These issues underscore the significance of NO_2_ detection.^[^ ^3^, ^4^, ^5^, ^6^, ^7^, ^8^, ^9^ ^]^

Among the myriad kinds of NO_2_ sensors, metal oxide (MOX)‐based gas sensors are favored due to their low cost, ease of fabrication, high response, and low detection limit.^[^ ^3^, ^5^ ^]^ However, their applications are limited by poor selectivity and high power consumption because of the susceptibility to environmental changes and the requirement of elevated operating temperatures.^[^ ^9^, ^10^, ^11^, ^12^, ^13^ ^]^ Gas selectivity is usually defined as the phenomenon of the response to the target gas being higher than that to interfering gases.^[^ ^11^ ^]^ The foundation of the sensing mechanism of the MOX‐based gas sensor lies in the interface charge transfer between metal oxides and gas molecules, which depletes or increases the charge carriers, leading to resistance changes. This is named as the space charge model.^[^ ^3^, ^13^ ^]^ Therefore, various gases could activate the response of the sensor, which inhibits its ability of recognizing the target gas from a gas mixture environment. Hence, a lack of gas selectivity is a highly recognized shortage of MOX‐based gas sensors and many researchers have focused on improving the selectivity.^[^ ^10^, ^11^, ^12^, ^13^, ^14^ ^]^ Besides, humidity is a common interference in real atmospheres while many studies reported that the gas sensing performance of MOX is highly influenced by humidity.^[^ ^15^, ^16^ ^]^ This will inhibit the real applications of the MOX‐based gas sensors and improving their humidity resistance is an important goal.

Recently, defect engineering is receiving increasing attention in surface‐related applications such as gas sensing and catalysis.^[^ ^3^, ^5^, ^12^, ^13^, ^14^, ^15^, ^16^, ^17^, ^18^, ^19^, ^20^, ^21^ ^]^ Numerous studies have reported that oxygen vacancy (Ov) improved the sensing performance of MOX‐based gas sensors.^[^ ^3^, ^5^, ^12^, ^13^, ^19^, ^20^, ^21^, ^22^, ^23^ ^]^ First, O*v* improves the gas‐sensitive behavior by providing more oxygen adsorption sites and promoting the formation of chemisorbed oxygen species.^[^ ^3^, ^5^, ^12^, ^13^ ^]^ Second, O*v* causes lattice distortions, chemical bond reconstruction, and thus changes the energy band structures of MOX. New energy states are generated in the forbidden band and sometimes the band gap of the MOX is narrowed, which decreases the energy barrier and increases the carrier mobility, hence enhances interfacial charge transfer, that is, higher sensing response.^[^ ^3^, ^20^ ^]^ Besides, it is reported that controlling the O*v* of MOX allows specific surface adsorptions and therefore regulates the gas selectivity. Increased O*v* can enhance the adsorption of the MOX toward oxidizing gases.^[^ ^21^ ^]^ Several studies have reported that O*v* leads to the formation of more active sites to adsorb NO_2_, and the adsorption capacity of NO_2_ on surfaces with O*v* is much stronger than that on non‐defective surfaces.^[^ ^3^, ^19^ ^]^ Additionally, O*v* has been reported to enhance the reductivity of the MOX in the field of photocatalysis.^[^ ^24^, ^25^ ^]^ From this finding, it is reasonable for us to propose that increased O*v* should enhance the selectivity of the MOX toward oxidizing gases like NO_2_. Overall, it is promising to improve the response and selectivity of a MOX‐based NO_2_ sensor by increasing the O*v* of the MOX.

TiO_2_ has received extensive attention in the fields of gas sensing, photocatalysis, and biosensing due to its high oxidation activity, high chemical inertness, and stable catalytic properties. Notably, black TiO_2_ outperforms white TiO_2_ in areas like photocatalysis and biomedicine.^[^ ^26^, ^27^ ^]^ For example, the unique defect structure of black TiO_2_ suppresses electron–hole recombination, enhancing the separation efficiency of photogenerated charge carriers. This promotes the generation of reactive oxygen species, which are utilized to improve the efficacy of photodynamic cancer therapy and to achieve immunomodulation.^[^ ^28^ ^]^ The narrowed bandgap of black TiO_2_ extends the light absorption from the UV region (3–3.2 eV) into the visible and near‐infrared (NIR) ranges. This enhances absorption of NIR (700–950 nm) and (1000–1700 nm) light, enabling precise photothermal therapy to destroy tumor cells such as melanoma and breast cancer cells using NIR irradiation.^[^ ^26^ ^]^ The oxygen vacancies of black TiO_2_ enhance photothermal conversion efficiency. Researchers utilized this property of black TiO_2_ to create a smart temperature‐regulating system by combining the black TiO_2_ with white TiO_2_.^[^ ^29^ ^]^ This efficient photothermal conversion capability (significantly superior to conventional white TiO_2_) is utilized to develop antibacterial functional materials. ZnO/black TiO_2‐x_ heterojunction implants eradicate bacteria through photothermal effects, achieving efficient antibacterial, antibiofilm, and immunomodulatory functions.^[^ ^30^ ^]^ The oxygen vacancies and surface hydroxyl groups of black TiO_2_ significantly boosts the separation efficiency of photogenerated carriers and catalyzes water hydrolysis to produce protons. The resulting proton concentration gradient then drives the aggregation and oscillation of B‐TiO_2_@N/Au nanorobots via diffusiophoresis or electroosmotic flow, offering a groundbreaking tool for neuromodulation and biomimetic systems.^[^ ^27^ ^]^ All of the above works demonstrated unique advantages of black TiO_2_ in areas such as photodynamic therapy,^[^ ^26^, ^28^, ^30^ ^]^ constructing smart temperature‐regulating materials,^[^ ^29^ ^]^ and driving microrobots.^[^ ^27^ ^]^ However, it has been rarely used in gas sensors based on our knowledge.

In this work, black and white TiO_2_ burr‐like nanorods were obtained by controlling the annealing atmospheres. The gas‐sensing performance of the black TiO_2_ to NO_2_ and several other gases were compared with the common white TiO_2_ in terms of response magnitudes, response types, selectivity, detection limit and humidity immunity. A room‐temperature‐operated NO_2_ sensor with ultrahigh selectivity and humidity resistance is developed using black TiO_2_. A wireless portable equipment has been developed and its capabilities in identifying NO_2_ from a mixed atmosphere and recognizing environmental differences in practical scenarios have been examined. Through material characterization, response comparison, gas‐TiO_2_ interactions study and density functional theory (DFT) calculation, novel mechanisms are presented for the observed various response types and enhanced sensing performance.

## 2 Results and Discussion

### 2.1 Characterizations

**Figure 1:**
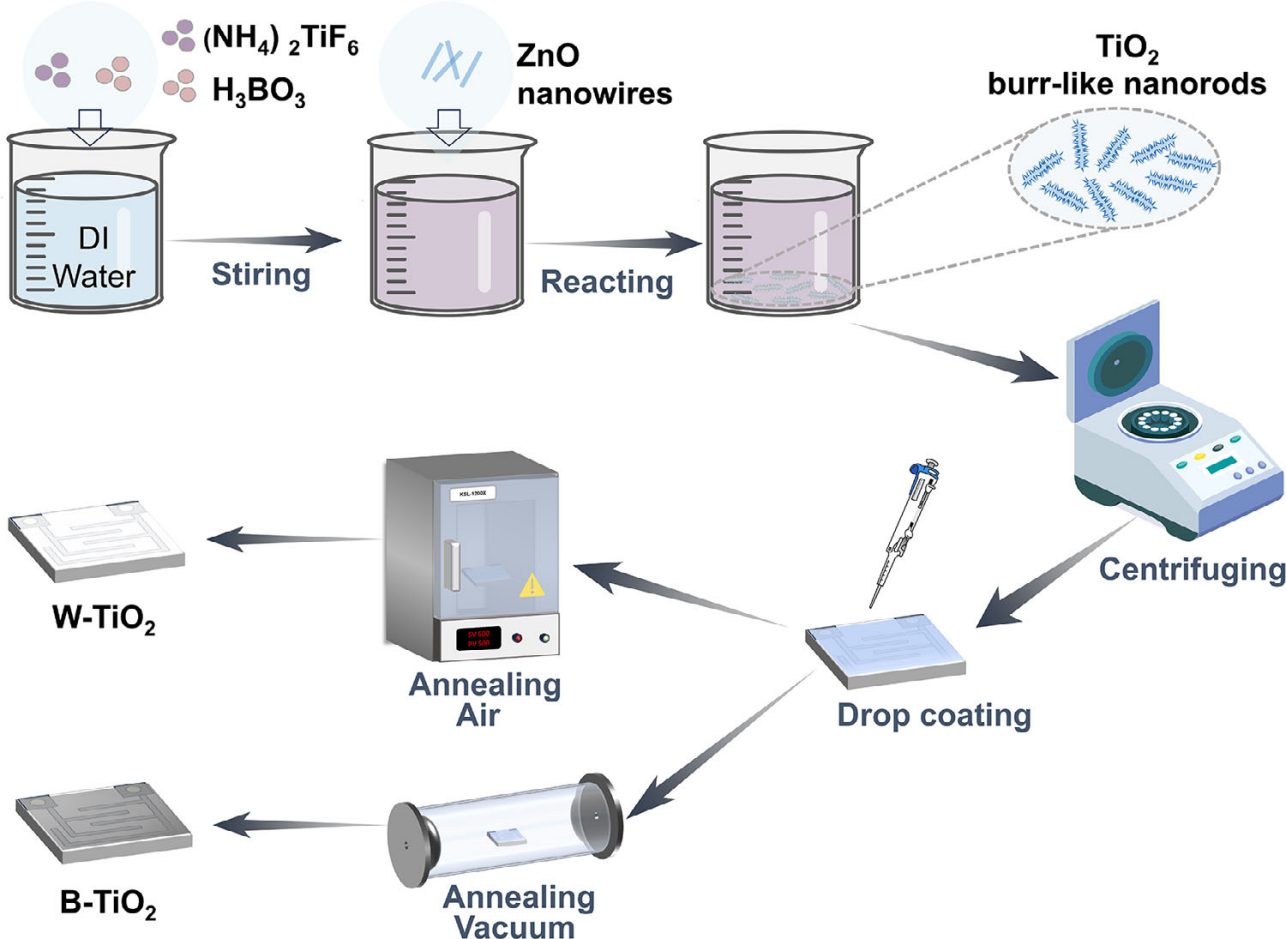
Synthesis of TiO_2_ burr‐like nanorods using the liquid precipitation method.

The schematic for the preparation of the TiO_2_‐based device is illustrated in **Figure** **1**. The TiO_2_ burr‐like nanorods were fabricated by a liquid precipitation method. Then, the TiO_2_ was drop‐coated on ceramic electrodes, followed by annealing in two different atmospheres. TiO_2_ with varying O*v* contents was obtained (see the Experimental Section for details). The TiO_2_ annealed in air is white and denoted as W‐TiO_2_. The TiO_2_ annealed in vacuum is black and denoted as B‐TiO_2_.

**Figure 2:**
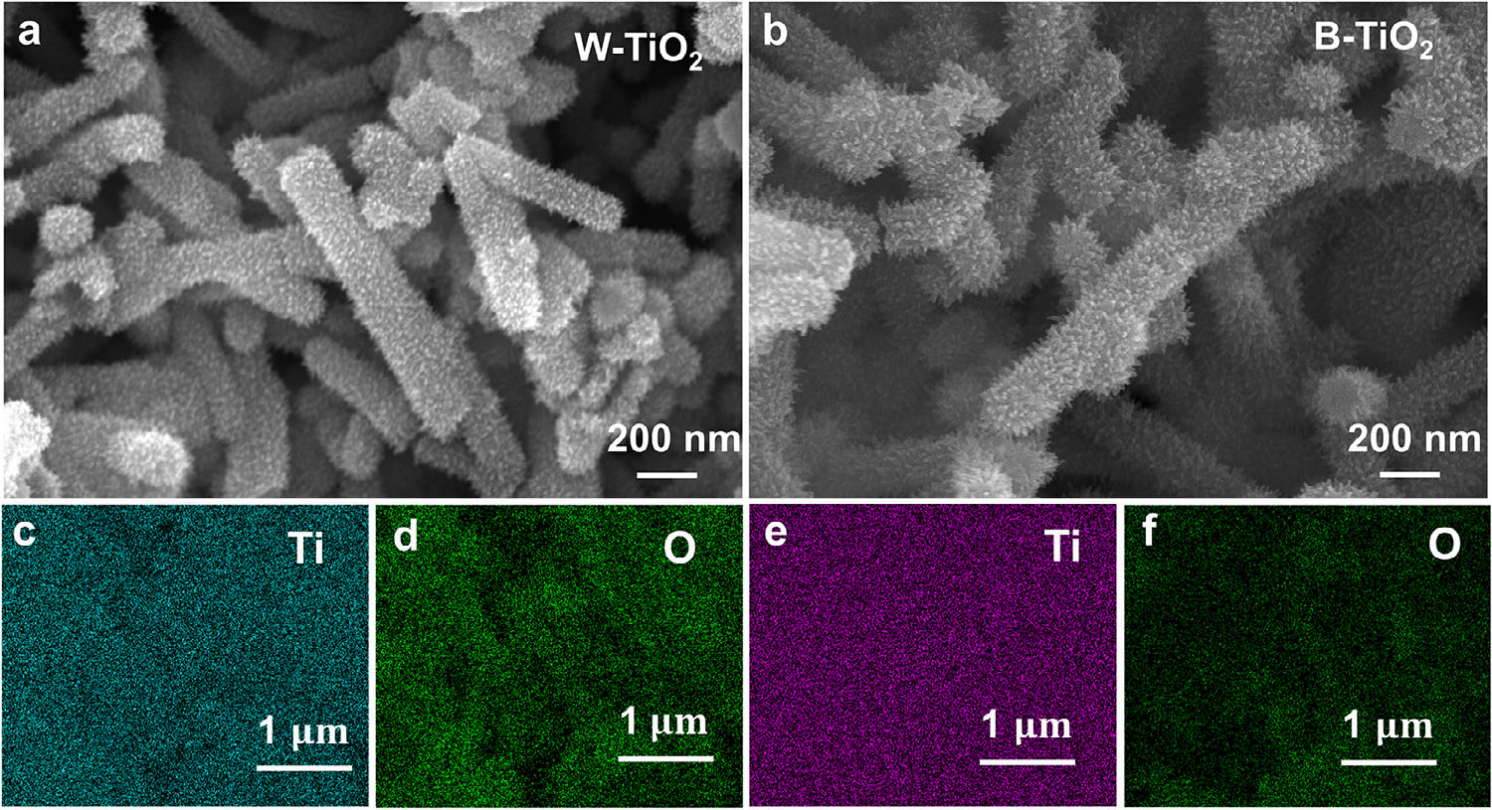
SEM images of a) W‐TiO_2_, b) B‐TiO_2_. EDS mapping images of c,d) W‐TiO_2_ and e,f) B‐TiO_2_.

The field emission scanning electron microscope (FESEM) images of W‐TiO_2_ and B‐TiO_2_ are displayed in **Figure** **2**a,b separately, which show that both W‐TiO_2_ and B‐TiO_2_ are burr nanorods with diameters ranging from 140 to 250 nm, identical in morphology. The energy dispersive spectroscopy (EDS) mapping data of W‐TiO_2_ (Figure 2c,d) and B‐TiO_2_ (Figure 2e,f) show a uniform distribution of Ti and O elements. The oxygen content in B‐TiO_2_ (Figure 2f) is less than that in W‐TiO_2_ (Figure 1d). This difference might be attributed to the creation of a large number of O*v* under vacuum annealing conditions.

**Figure 3:**
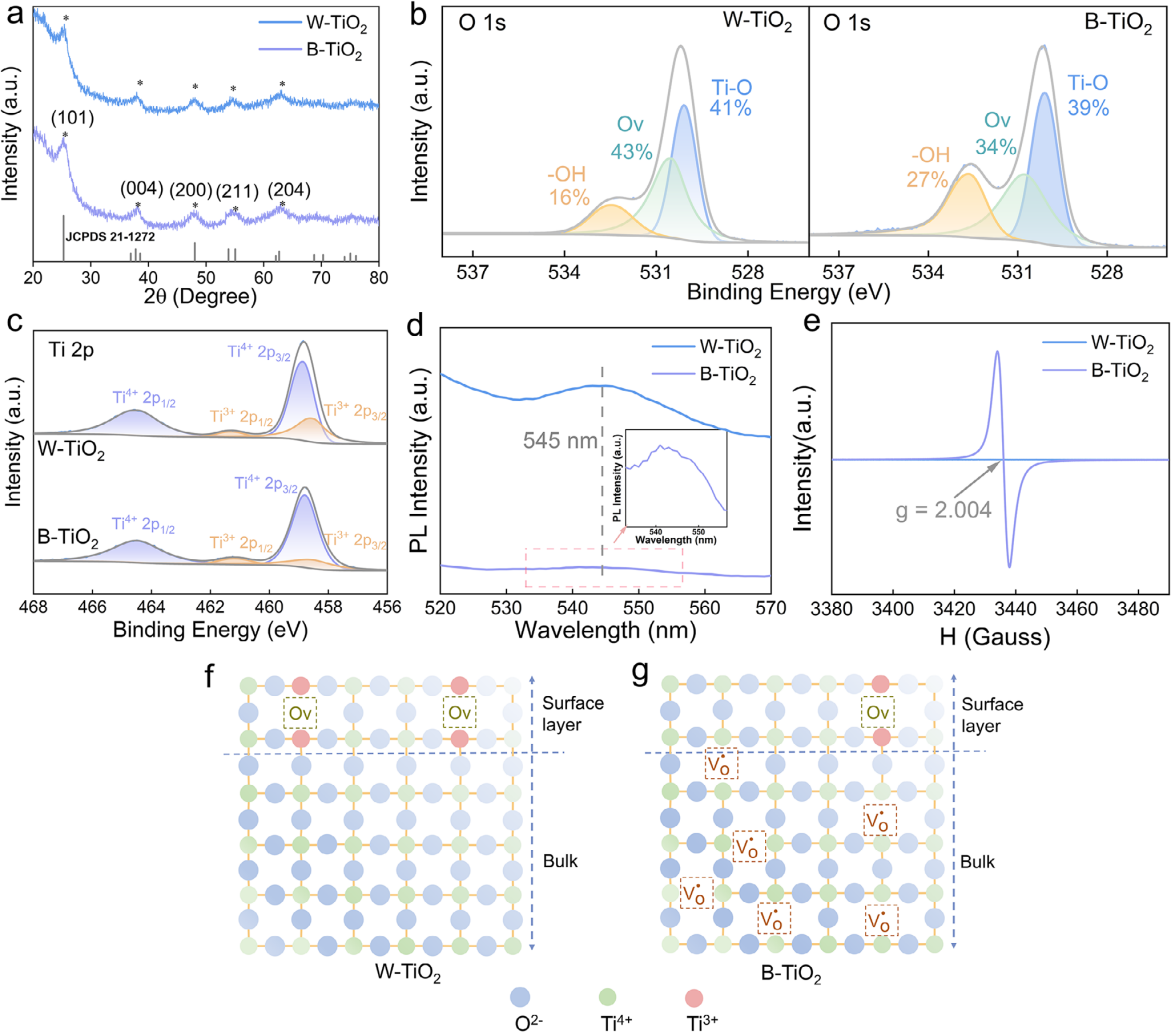
a) XRD patterns. b) O 1s XPS spectra. c) Ti 2p XPS spectra. d) PL spectra. e) EPR spectra. The schematic diagram of O*v* distribution in f) W‐TiO_2_ and g) B‐TiO_2_.

The X‐ray diffraction (XRD) patterns of the W‐TiO_2_ and B‐TiO_2_ are shown in **Figure** **3**a. All diffraction peaks of the XRD patterns of both samples represent the anatase TiO_2_ (JCPDS Card No. 21–1272) and no additional peaks were observed.^[^ ^28^ ^]^ X‐ray photoelectron spectroscopy (XPS) and photoluminescence (PL) measurements were performed to compare the O*v* between the W‐TiO_2_ and B‐TiO_2_. The main constituent elements of W‐TiO_2_ and B‐TiO_2_ are Ti, O, and C as shown by the XPS patterns in Figure S1 (Supporting Information). The deconvolution of O 1s spectra for W‐TiO_2_ and B‐TiO_2_ gives three peaks corresponding to different oxidation states of O (Figure 3b): lattice oxygen (Ti‐O), defective oxygen (O*v*), and the surface‐adsorbed hydroxyl groups (─OH).^[^ ^31^, ^32^ ^]^ Compared with the W‐TiO_2_, the hydroxyl groups increased, the lattice oxygen and defective oxygen decreased in the B‐TiO_2_. Figure 3c shows the deconvolution XPS spectra of Ti 2p of W‐TiO_2_ and B‐TiO_2_. The peaks at 458.86 and 464.47 eV correspond to Ti^4+^ 2p_3/2_ and Ti^4+^ 2p_1/2_, respectively. The shoulder peaks ≈458.53 and 461.13 eV correspond to Ti^3+^ 2p_3/2_ and Ti^3+^ 2p_1/2_, respectively.^[^ ^33^ ^]^ The XPS spectra of Ti 2p show that the ratio of Ti^4+^: Ti^3+^ is 75.66: 24.34 in W‐TiO_2_, and 81.33: 18.67 in B‐TiO_2_. Compared to W‐TiO_2_, B‐TiO_2_ exhibits an increased Ti^4+^ and a decreased Ti^3+^. Since the Ti^4+^ can be reduced to Ti^3+^ by capturing electrons from the O*v*, the decreased Ti^3+^ in the B‐TiO_2_ indicates the decreased O*v*.^[^ ^34^ ^]^ Therefore, the increased Ti^4+^: Ti^3+^ in B‐TiO_2_ further confirms that B‐TiO_2_ contains less O*v* than W‐TiO_2_ does, as shown by Figure 3b. The PL spectra of W‐TiO_2_ and B‐TiO_2_ are shown in Figure 3d. Both W‐TiO_2_ and B‐TiO_2_ exhibit a broad emission peak at 545 nm, which reflects the surface O*v*.^[^ ^35^, ^36^ ^]^ The peak of the B‐TiO_2_ is weaker than that of the W‐TiO_2_, further indicating that B‐TiO_2_ contains less surface O*v* than W‐TiO_2_ does. Meanwhile, the PL intensity of B‐TiO_2_ is significantly lower than that of W‐TiO_2_, indicating that the recombination rate of photogenerated electrons and holes is greatly suppressed in B‐TiO_2_.^[^ ^37^ ^]^ This suppressed recombination further confirms the aforementioned claim that the B‐TiO_2_ contains more OH groups than the W‐TiO_2_ does (Figure 3b) because photogenerated holes are easily trapped by chemisorbed surface OH groups, generating OH radicals and thus inhibiting their radiative complexation.^[^ ^38^ ^]^

Overall, both XPS and PL results indicate that W‐TiO_2_ contains more surface O*v* and fewer OH groups than B‐TiO_2_ does. However, the aforementioned EDS results show that W‐TiO_2_ contains less O*v* than B‐TiO_2_ does. Addressing this inconsistency, we further employed electron paramagnetic resonance (EPR) spectroscopy. As show by Figure 3e and B‐TiO_2_ shows a strong EPR signal at *g* = 2.004, which is identified as an electron trapped in an O*v*.^[^ ^39^, ^40^ ^]^ This peak is hardly observed in W‐TiO_2_. This confirms that B‐TiO_2_ contains more O*v* than W‐TiO_2_ does as the aforementioned EDS result claimed. The contradiction between the XPS, PL, and the EPR, EDS could be attributed to the differences among these characterization techniques and the difference in states of the oxygen vacancies as explained in the following. There are two states of the O*v* formed on the TiO_2_ surface, O*v* with two electrons (*V*_O_^⚫⚫^) or O*v* without electrons (*V*_O_), neither of which gives EPR signals. Only the singly‐ionized O*v* with one electron (*V*_O_^⚫^) can be detected by EPR. The peak at *g* = 2.004 is generally attributed to these *V*_O_^⚫^ in the bulk.^[^ ^39^ ^]^ However, XPS and PL generally reflect the surface states. In summary, both W‐TiO_2_ and B‐TiO_2_ contain a few surface O*v*, with little difference between them, as demonstrated by the XPS and PL. However, B‐TiO_2_ contains significantly more bulk *V*_O_^⚫^ than W‐TiO_2_ does. The O*v* distributions of W‐TiO_2_ and B‐TiO_2_ are shown schematically in Figure 3f,g.

### 2.2 Gas Sensing Performance

The sensing performance of sensors based on W‐TiO_2_ and B‐TiO_2_ to NO_2_ and several other common interfering gases at room temperature was studied (the measurement details are described in the Experimental Section).

**Figure 4:**
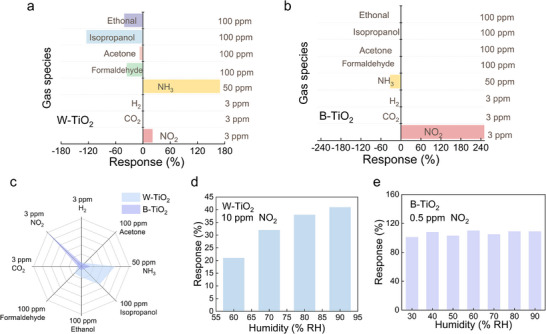
Gas sensing responses of a) W‐TiO_2_ and b) B‐TiO_2_. c) Radar chart of the response and selectivity of W‐TiO_2_ and B‐TiO_2_. d) Responses of W‐TiO_2_ to 10 ppm NO_2_ at various humidities. e) Responses of B‐TiO_2_ to 0.5 ppm NO_2_ at various humidities.

**Figure** **4**a,b summarizes the response of W‐TiO_2_ and B‐TiO_2_ to NO_2_ and several common gases including ethanol, isopropanol, acetone, formaldehyde, carbon dioxide (CO_2_), ammonia (NH_3_), and hydrogen (H_2_), respectively. The responses of W‐TiO_2_ and B‐TiO_2_ to 3 ppm NO_2_ are 20.9% and 250%, respectively. The B‐TiO_2_ exhibits a 12‐times higher response than W‐TiO_2_ does. Besides, the B‐TiO_2_ improves selectivity to NO_2_ remarkably. As shown in Figure 3a, the W‐TiO_2_ showed responses of −41%, −124%, −7%, and −35% to 100 ppm ethanol, isopropanol, and formaldehyde, respectively, 169% to 50 ppm NH_3_, and no response to 3 ppm CO_2_ and H_2_. The B‐TiO_2_ suppresses the responses to all the above 7 interference gases, showing almost no response to ethanol, isopropanol, acetone, formaldehyde, as well as CO_2_ and H_2_, and decreasing the response to 50 ppm NH_3_ by 5 times to only 32%. In this way, the B‐TiO_2_ sensor exhibits ultra‐high selectivity toward NO_2_, which is more intuitively reflected in the radar chart in Figure 4c (the data in the graph are absolute values of the response magnitudes).

Humidity is a common interference during NO_2_ detection.^[^ ^15^, ^16^ ^]^ The humidity resistance was examined by comparing the NO_2_ sensing performance of W‐TiO_2_ and B‐TiO_2_ at different humidity levels. The corresponding real‐time response curves are provided in Figure S2 (Supporting Information) and the response magnitudes are summarized in Figure 4d,e. The response of W‐TiO_2_ to NO_2_ increases as the humidity increases from 60% to 90% (Figure 4d). The resistance of the W‐TiO_2_ is too large to be measured by our equipment when the humidity is less than 60%. In contrast, the response of B‐TiO_2_ to NO_2_ is unaffected by humidity in the atmosphere within the relative humidity range of 30–90% (Figure 4e). The B‐TiO_2_ exhibits remarkably improved humidity immunity during NO_2_ detection. The remarkable improvements in gas selectivity and humidity resistance indicate the robust potential of this NO_2_ sensor for practical applications.

**Figure 5:**
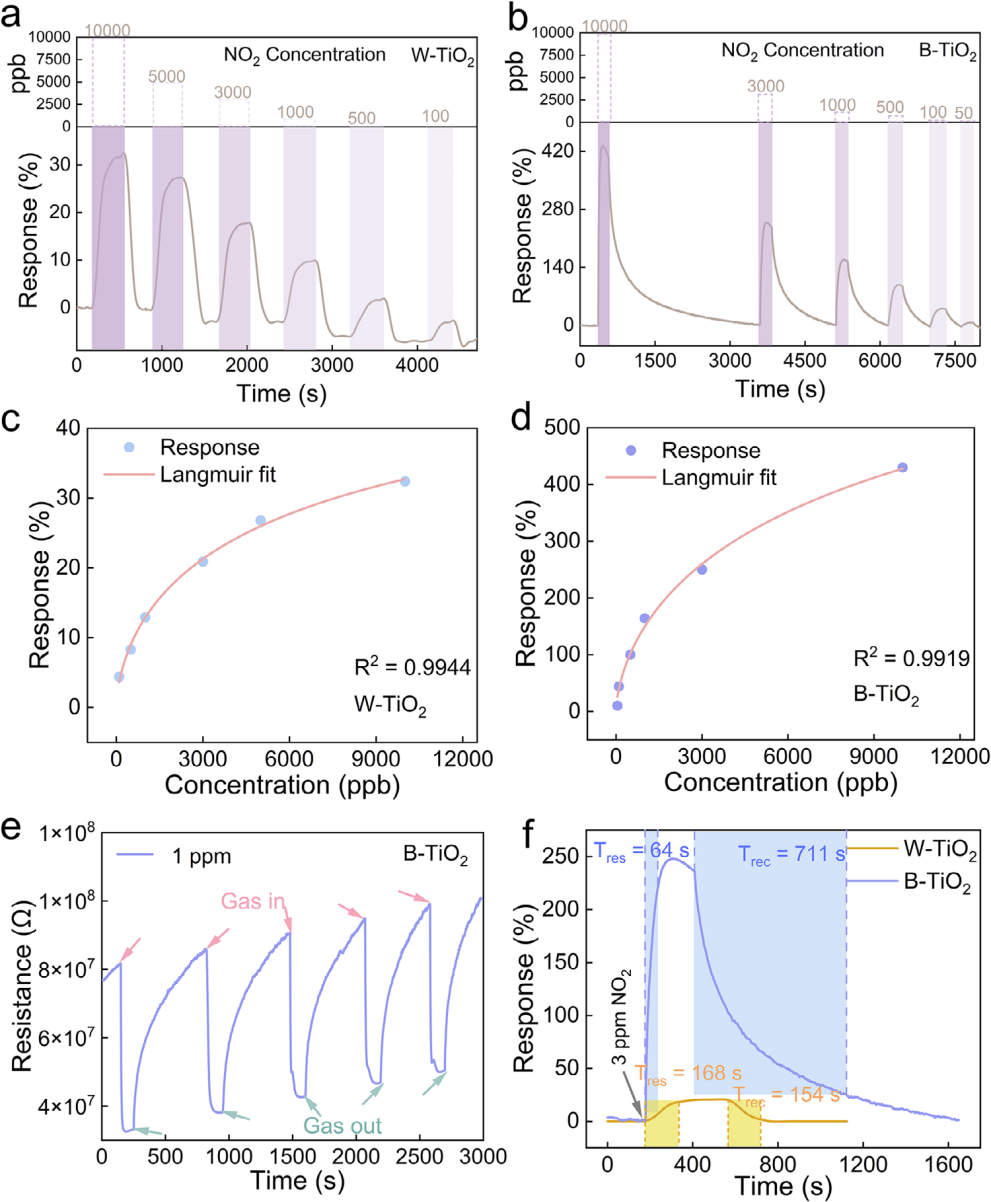
Real‐time sensing curves of a) W‐TiO_2_ and b) B‐TiO_2_ to NO_2_ at various concentrations. Fitting relationship between the response and NO_2_ concentration of c) W‐TiO_2_ and d) B‐TiO_2_. e) Sensing reproductivity of B‐TiO_2_ to 1 ppm NO_2_. f) Response and recovery times of W‐TiO_2_ and B‐TiO_2_ to 3 ppm NO_2_.

The dynamic response curves to NO_2_ of various concentrations of the W‐TiO_2_ and B‐TiO_2_ to NO_2_ are shown in **Figure** **5**a,b, respectively. Both responses increase as the NO_2_ concentration increases. Compared to W‐TiO_2_, B‐TiO_2_ significantly enhances the response to NO_2_ by more than 10 times at all measured concentrations. For example, the response of the W‐TiO_2_ to 10 ppm NO_2_ is 32.4%, while the corresponding response of B‐TiO_2_ reaches up to 430%. At lower concentrations like 100 ppb, the response of W‐TiO_2_ is 4.4%, while the response of B‐TiO_2_ reaches 44%. Besides, the B‐TiO_2_ exhibits a 10% response to 50 ppb NO_2_, demonstrating its ability of low concentration NO_2_ detection. The response‐concentration relationships of the W‐TiO_2_ and B‐TiO_2_ are fitted with the Langmuir isotherm as shown in Figure 5c,d, as expressed in Equations (1) and (2):

(1) $$\mathrm{Response}\left(\%\right)=\frac{0.187C{\left(ppb\right)}^{0.63}}{1+0.003C{\left(ppb\right)}^{0.63}}\times 100\%$$

(2) $$\mathrm{Response}\left(\%\right)=\frac{1.984C{\left(ppb\right)}^{0.63}}{1+0.002C{\left(ppb\right)}^{0.63}}\times 100\%$$

where C is the concentration in ppb. The corresponding correlation coefficients *R* ^2^ were 0.9944 and 0.9919, respectively, indicating that the sensor can be used for NO_2_ concentration monitoring. Furthermore, the B‐TiO_2_ maintains stability over 5 cycles for 1 ppm NO_2_ (Figure 5e), indicating that the sensor has good repeatability. 50 cycle‐sensing performance to 500 ppb NO_2_ was obtained by repeating the measurements continuously, which lasted for ≈14 h. The result is shown in Figure S3 (Supporting Information), further confirming the repeatability of the device. Figure 5f shows the response time (*T*_res_) and recovery time (*T*_rec_) of W‐TiO_2_ and B‐TiO_2_ to 3 ppm NO_2_. The response time of B‐TiO_2_ (64 s) is much shorter than that of W‐TiO_2_ (168 s). However, the B‐TiO_2_ exhibits a longer recovery time than W‐TiO_2_ does, which may be attributed to its higher concentration of bulk O*v*, making the desorption of NO_2_ gas molecules more difficult.^[^ ^41^ ^]^

The sensing performance of the B‐TiO_2_‐based sensor provided in this work is compared to some previously reported NO_2_ sensors as shown in **Table** **1**. The comparison reveals that MOX‐based sensors generally rely on thermal excitation, optical excitation, or composite materials, as shown in entries 1–12 of the table. And the room temperature‐operated gas sensors based on pure metal oxides usually exhibit a low response magnitude, as shown in entries 13–15 of the table. In contrast, the B‐TiO_2_‐based sensor developed here demonstrates advantages in response time, response magnitude, and detection limit. The most important thing is that, the B‐TiO_2_‐based sensor exhibits a remarkably enhanced selectivity to NO_2_ and excellent humidity immunity.

**Table 1:** Comparison table of NO_2_ sensing characteristics of B‐TiO_2_ versus previously reported MOX‐based NO_2_ sensors.

Material	*T* ^a)^ [°C]	Con. ^b)^ [ppm]	Response type	Response magnitude	*T*_res_ [s]	LOD^c)^	Ref.
TiO_2_	500	2	n	*R*_g_/*R*_a_ = 2.3	90	2 ppm	[42]
ZnO‐TiO_2_	250	20	n	(*R*_g_ − *R*_a_)/*R*_a_ × 100% = 4	120	2 ppm	[43]
Cr‐TiO_2_	500	10	n	*R*_a_/*R*_g_ = 3.2	180	10 ppm	[44]
α‐MoO_3_	125	1	n	(*R*_g_ − *R*_a_)/*R*_a_ × 100% = 314.5	1113	50 ppb	[45]
V_2_O_5_	150	100	n	(*R*_g_ − *R*_a_)/*R*_a_ × 100% = 13	4	10 ppm	[46]
TiO_2_	RT^d)^/UV^e)^	100	n	(*R*_g_ − *R*_a_)/*R*_a_ × 100% = 1.3	100	100 ppm	[47]
TiO_2_/graphene	RT/UV	1.75	p	*R*_a_/*R*_g_ = 3.14	35	70 ppb	[48]
PrGO‐TiO_2_	RT/UV	100	n	*R*_a_ − *R*_g_/*R*_a_ × 100% = 35.6	75	10 ppm	[49]
TiO_2_ QDs/MoSe_2_	RT/UV	100	p	*R*_a_/*R*_g_ = 4.16	9.7	1 ppm	[50]
ZnO‐TiO_2_‐PANI	RT	20	p	(*R*_g_ − *R*_a_)/R_a_ = 412	87	1 ppm	[51]
TiO_2_‐ppy‐MoO_x_	RT	1	n	*R*_g_/*R*_a_ = 11.96	9	1 ppb	[52]
V_Cl_‐Cs_2_AgInCl_6_/TiO_2_	RT	1	n	*R*_g_/*R*_a_ = 7.26	38	20 ppb	[53]
In_2_O_3_	RT	50	n	(*R*_g_ − *R*_a_)/*R*_a _ × 100% = 219	89	500 ppb	[54]
Mn_3_O_4_	RT	40	n	(*R*_g _− *R*_a_)*/R*_a_ × 100% = 62	116	5 ppm	[55]
TiO_2_	RT	1	n	(*R*_g_ − *R*_a_)/*R*_a _× 100% = 18.5	75	250 ppb	[56]
TiO_2_	RT	3	p	(*R*_a_ − *R*_g_)/*R*_g_ × 100% = 250 *R*_a_/*R*_g_ = 3.5	69	50 ppb	This work
		10		(*R*_a_ − *R*_g_)/*R*_g_ × 100% = 430 *R*_a_/*R*_g_ = 5.3	38		

^a)^ Operation temperature;

^b)^ NO_2_ Concentration;

^c)^ Experimental detection limit;

^d)^ Room temperature;

^e)^ UV illumination.

**Figure 6:**
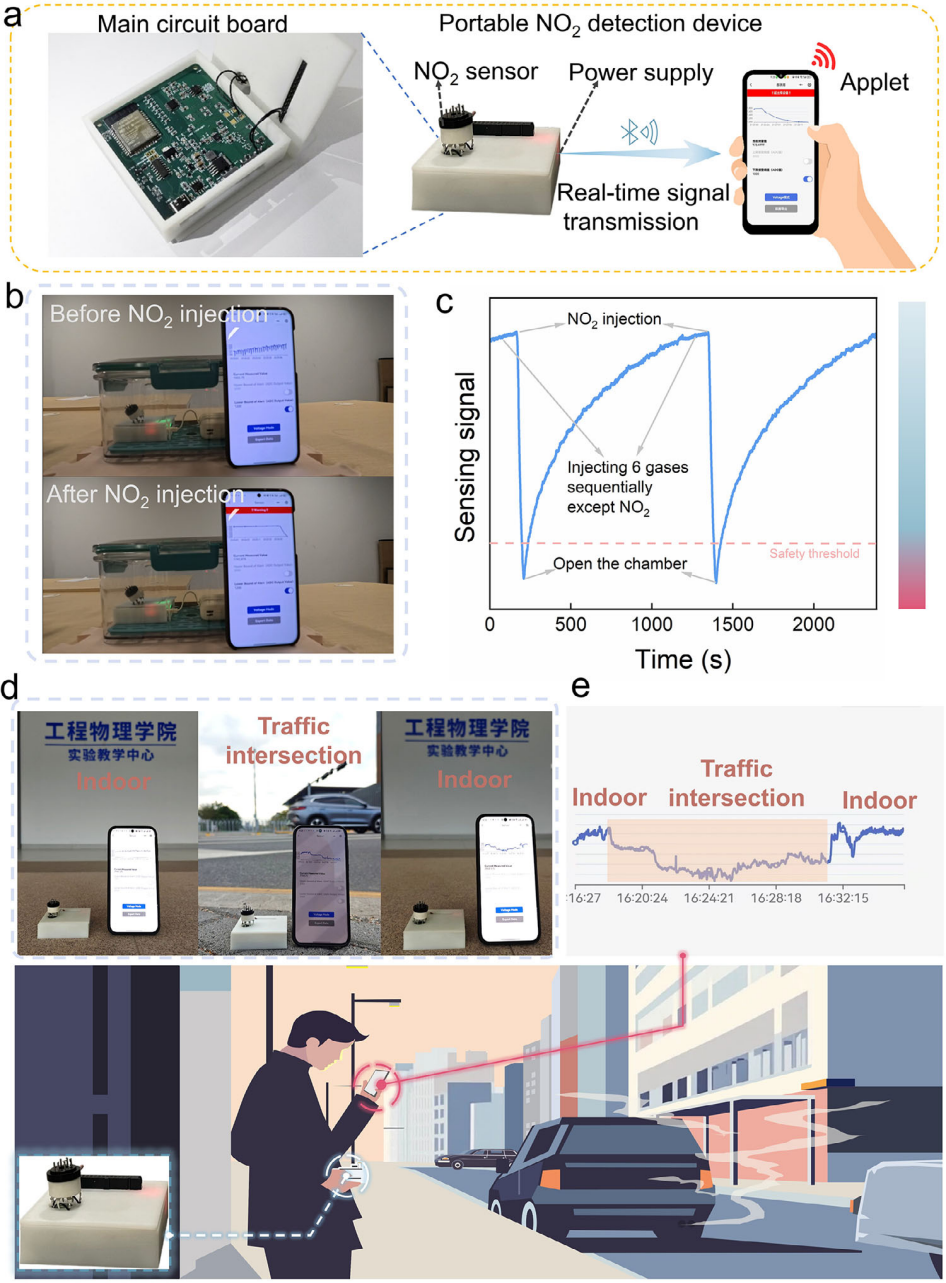
a) Schematic of the portable NO_2_ detection equipment. b) Photograph of NO_2_ detection process using the portable equipment, and c) the corresponding real‐time response curve to NO_2_ in a mixed atmosphere. d) Photograph of real‐environment identification process using the portable equipment and e) the corresponding real‐time response curve in different environments.

A portable wireless NO_2_ sensor equipment was developed and used for on‐field NO_2_ detection in gas mixtures and real scene identification (**Figure** **6**a). The sensor box consists of a B‐TiO_2_‐based sensor, a power supply, a main circuit board (integrated microcontroller (MCU) module, a low‐power Bluetooth transceiver module (BLE)), and encapsulation. The BLE wirelessly transmits the real‐time signal collected by the sensor box to the smartphone. A self‐developed applet is integrated into the smartphone. The real‐time output value of the analogue‐to‐digital converter, which corresponds to the real‐time voltage of the B‐TiO_2_‐based sensor, is displayed on the interface of the applet in the screen of the smartphone. The smartphone would trigger an alarm when the concentration of the target gas exceeds a dangerous level, which is set in the applet in the smartphone. The measurement progress of NO_2_ sensing performance in a mixed atmosphere is illustrated in Figure 6b and Video S1 (Supporting Information). The sensor box is placed in a sealed chamber where various atmospheric environments could be simulated. The formaldehyde, ethanol, isopropanol, acetone, CO_2_, and H_2_ gases are injected into the sealed chamber sequentially, while the output curve shown on the smartphone shows no significant fluctuations. This demonstrates that the sensor shows no response to the above 6 gases again, as well as their mixtures. Now, the atmosphere in the chamber is a composite of the above 6 gases and the background air. Then, NO_2_ is injected into the chamber which contains a composite of the above 6 gases and the background air, a decreasing signal appears immediately in the output curve shown in the screen of the smartphone. This demonstrates the capability of the portable NO_2_ detection device in distinguishing NO_2_ from other gases in mixed atmospheres experimentally. Upon the NO_2_ concentration exceeding the set dangerous concentration, the mobile phone alarm is triggered. Then, upon opening the sealed chamber, the NO_2_ disperses, the signal recovers, and the alarm stops. This phenomenon once again confirms that the device possesses recovery performance even in complex mixed gas environments, and its performance remains unaffected by the above 6 gases. Then, this measurement procedure was repeated, and the result proved the repeatability of this portable equipment for NO_2_ detection in a complex mixed atmosphere. The corresponding response curve to NO_2_ in the mixed atmosphere is shown in Figure 6c. Furthermore, the equipment was used to detect the environmental difference between the traffic intersection and the indoor environment (Figure 6d). The traffic intersection contains more vehicles as well as more NO_2_ than the indoor does. The real‐time sensing curve is shown in Figure 6e, showing a decrease in the signal upon entering the traffic intersection and recovering upon returning indoor. This demonstrates that the B‐TiO_2_‐based sensor could identify the environmental differences in real applications.

### 2.3 Sensing Mechanisms

Generally, the space charge model, which involves adsorption of gases and redox reactions between gas molecules and oxygen anions on the surface of the sensing material, is the commonly accepted sensing mechanism for MOX‐based gas sensors.^[^ ^3^, ^13^, ^57^, ^58^, ^59^ ^]^ The response type of the MOX‐based gas sensors is classified into n‐type and p‐type. In general, for common n‐type semiconductors (such as TiO_2_, ZnO, and SnO_2_), the resistance decreases in the presence of reducing gases and increases in the presence of oxidizing gases, which is termed n‐type response. For p‐type semiconductors (such as Cr_2_O_3_ and CuO), the resistance increases in the presence of reducing gases and decreases in the presence of oxidizing gases, which is termed a p‐type response.^[^ ^60^, ^61^ ^]^ The response type is usually consistent with the semiconductor type of the MOX.^[^ ^60^ ^]^

TiO_2_ generally exhibits n‐type responses to gases.^[^ ^11^, ^62^, ^63^ ^]^ However, the W‐TiO_2_ exhibits p‐type responses to the oxidizing NO_2_ and the reducing ethanol, isopropanol, acetone, and formaldehyde, except NH_3_ (Figure S4, Supporting Information). The W‐TiO_2_ only displays an n‐type response to the reducing NH_3_ at room temperature. The B‐TiO_2_ exhibits p‐type response to both the oxidizing NO_2_ and the reducing NH_3_ (Figure S4, Supporting Information). The anomalous p‐type response and the different response types to the reducing gases indicate that the conductivity type of the MOX is not the only factor influencing the response type. The sensing mechanisms should be different and correlated with the gas species, which cannot be covered by the space charge model mentioned above.

**Figure 7:**
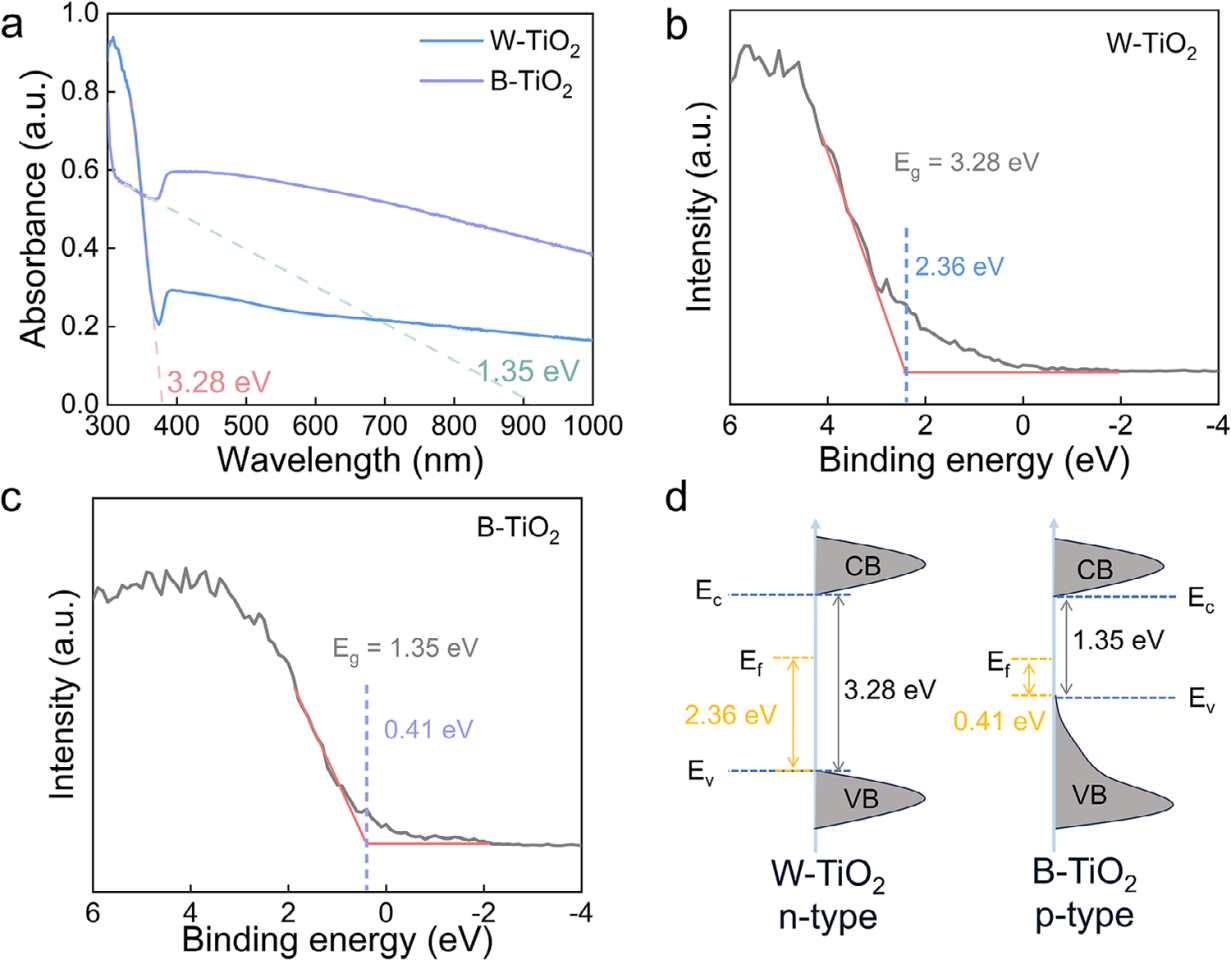
a) UV–vis absorption spectra of W‐TiO_2_ and B‐TiO_2_, respectively. VB‐XPS spectra of b) W‐TiO_2_ and c) B‐TiO_2_. d) The band energy diagram of W‐TiO_2_ and B‐TiO_2_.

To explain the anomalous p‐type response of the W‐TiO_2_ and the B‐TiO_2_, their conduction types need to be clarified first. Hall effect measurement was conducted. Under a fixed magnetic field of 0.8 T, the Hall voltages of W‐TiO_2_ and B‐TiO_2_ films are negative and positive as shown in Table S1 (Supporting Information), respectively. This indicates that the majority charge carriers of W‐TiO_2_ and B‐TiO_2_ are electrons and holes, corresponding to n‐type and p‐type semiconductors, respectively. The surface conduction types of the W‐TiO_2_ and B‐TiO_2_ were studied by the UV–vis absorption spectra and valence band (VB)‐XPS spectra results. The UV–vis absorption spectra (**Figure** **7**a) show that the bandgap of W‐TiO_2_ is ≈3.28 eV, and the bandgap of B‐TiO_2_ narrows significantly to 1.35 eV. Figure 7b,c shows the VB‐XPS spectra of W‐TiO_2_ and B‐TiO_2_, respectively. The valence band maximum (VBM) of W‐TiO_2_ is located at 2.36 eV below the Fermi level (*E*_f_). The corresponding band gap of the W‐TiO_2_ is illustrated in Figure 7d, suggesting that its *E*_f_ is closer to the conduction band (CB), representing an n‐type semiconductor. In contrast, the VBM of B‐TiO_2_ is situated at 0.41 eV below the *E*_f_. The corresponding band gap of the B‐TiO_2_ is illustrated in Figure 7d, indicating that its E_f_ is closer to the VB, representing a p‐type semiconductor. For transition metal oxide semiconductors, the VB mainly comes from the 2p orbital of O and the CB mainly comes from the d orbital of the metal. The introduction of O*v* broadens the VB.^[^ ^64^, ^65^ ^]^ Compared with the W‐TiO_2_, the B‐TiO_2_ contains more bulk O*v* as demonstrated by the aforementioned EDS (Figure 2d,e) and EPR (Figure 3e). Therefore, the VBM of the B‐TiO_2_ is upward shifted and broadened, shortening the energy gap between the E_F_ and VB. This causes the E_F_ closer to the VB compared to the CB. Therefore, the p‐type response to both oxidizing NO_2_ and reducing NH_3_ of the B‐TiO_2_ is consistent with its semiconductor type, which does not contradict the widely used space charge model‐based sensing mechanism.

However, the n‐type W‐TiO_2_ exhibits anomalous p‐type responses to all the measured reducing and oxidizing gases, except reducing NH_3_. This indicates that the conventional space charge model‐based sensing mechanisms cannot adequately explain the experimental results. Therefore, it is necessary to investigate the gas sensing mechanisms of W‐TiO_2_ at room temperature.

The anomalous p‐type response of an n‐type MOX is receiving increasing attention in recent years and there are several speculations for the mechanisms. For single MOX‐based gas sensors, some studies claimed that the strong oxygen adsorption on the MOX led to the formation of a charge inversion layer on the surface region of the MOX, named a p‐type inversion layer. Therefore, the sensor exhibited an anomalous p‐type response.^[^ ^61^, ^66^ ^]^ Based on this inversion‐layer theory, theoretically the sensors should show p‐type responses to all gases. However, the W‐TiO_2_ showed an n‐type response to NH_3_ in this work. Besides, the aforementioned hall effect measurement and VB‐XPS have demonstrated that the W‐TiO_2_ is n‐type in both its bulk and surface. So, the inversion‐layer theory is not applicable to this work. Some studies attributed the anomalous p‐type response of an n‐type MOX to different surface reactions at different temperatures.^[^ ^67^, ^68^ ^]^ For example, n‐type Fe_2_O_3_ showed an anomalous p‐type response to NO_2_ at temperatures above 250 °C due to the reactions between NO_2_ and the adsorbed oxygen species, whereas a normal n‐type response below 250 °C due to the direct adsorption of NO_2_.^[^ ^67^ ^]^ However, their speculation for the anomalous p‐type response is not applicable to our study. First, ethanol, H_2_, and NO_2_ all activate n‐type response in their study, while the W‐TiO_2_ exhibits anomalous p‐type response to these gases. Second, the abnormal p‐type response to NO_2_ appears at high temperatures in their study, while it occurs at room temperature in this study. Another speculation is that the gas response is caused by the interaction between the gas and the water adsorbed on the MOX surface.^[^ ^15^, ^69^ ^]^ The self‐dissociation of water molecules in the water layer can lead to proton hopping behavior, which is called ion‐proton conduction.^[^ ^70^ ^]^ In the physically adsorbed water layer, the protons (H^+^) and hydroxide ions (OH^−^) diffuse due to the collision or self‐ionization of water molecules (Equation (3)). The released H^+^ protonates to another water molecule, forming hydrated hydrogen ions (H_3_O^+^) (Equation (4)). As water molecule adsorption intensifies, ion‐proton conduction progressively dominates over electronic conduction of the MOX. Therefore, the electrical conductivity of the W‐TiO_2_ is determined by ion‐proton conduction.

(3)

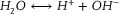



(4)

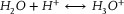




**Figure 8:**
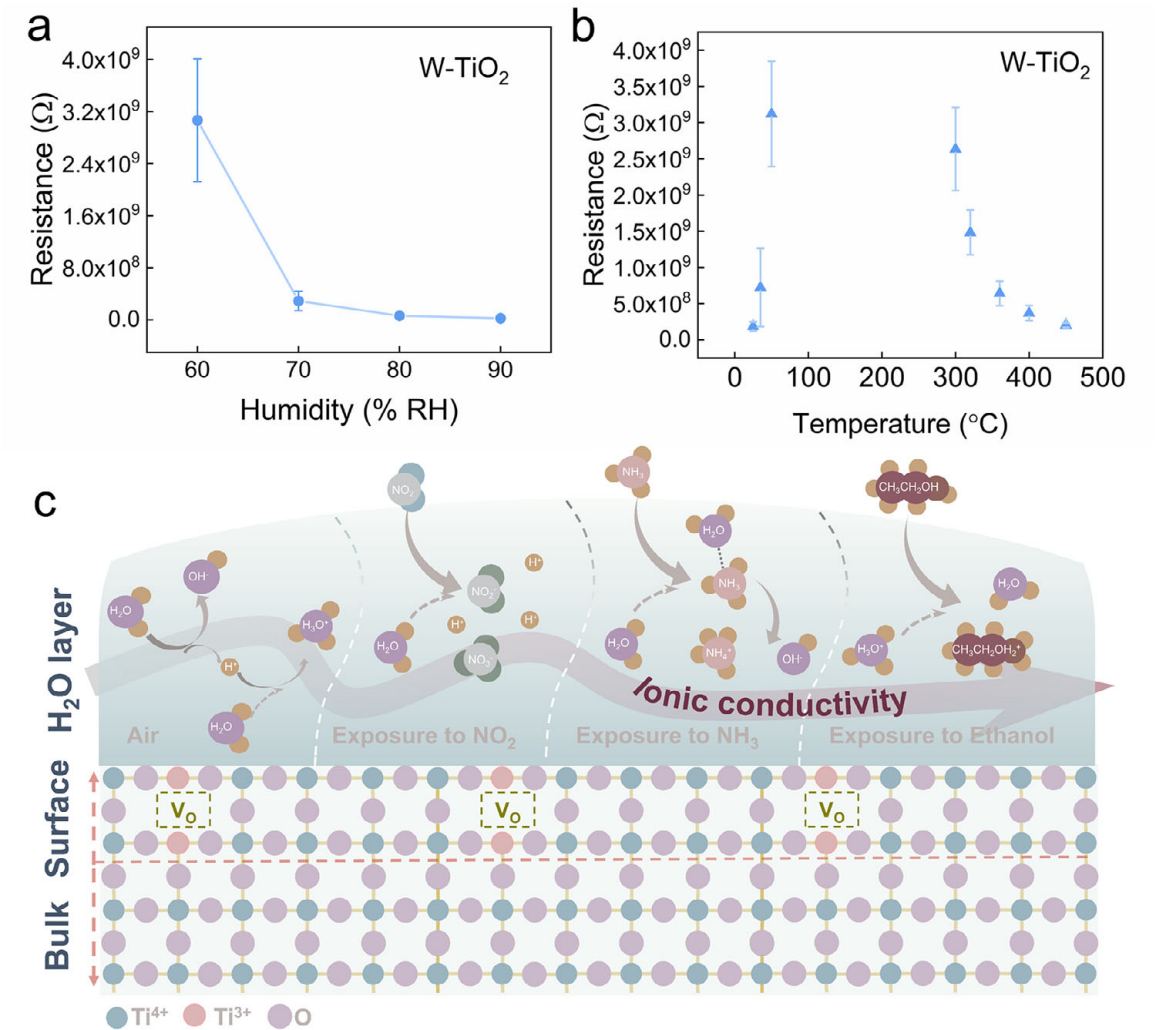
Resistance of W‐TiO_2_ as a function of a) humidity and b) temperature. c) Schematic representation of the sensing mechanisms to the measured gases of W‐TiO_2_.

In this case, the conductivity of W‐TiO_2_ should increase as the humidity increases due to the increased adsorption of water molecules. To verify this hypothesis, the conductivity of W‐TiO_2_ at various humidity levels was investigated. The result is shown in **Figure** **8**a which shows that the conductivity of W‐TiO_2_ increases as the humidity increases, which is consistent with the hypothesis claim. Besides, a dynamic cycling measurement of the conductivity of W‐TiO_2_ as the humidity changes back and forth for several times was conducted. The result is shown in Figure S5 (Supporting Information), which shows that the resistance always decreases as the humidity increases and increases as the humidity decreases, indicating excellent reversibility during the humidity fluctuations. These results validate the ion‐proton conduction model. Vice versa, in the case that ion‐proton conduction dominates the conductivity, the conductivity of W‐TiO_2_ should decrease if the adsorption of water molecules decreases. The temperature dependence of the resistance of the W‐TiO_2_ was investigated to confirm this. The result is shown in Figure 8b. The resistance of the W‐TiO_2_ first increases and then decreases as the temperature increases. The resistance of the W‐TiO_2_ between 100–300 °C is too large to be measured by our equipment. The decrease in resistance at higher temperatures is attributed to the thermal activation of carriers. The increase in resistance as temperature increases at lower temperatures could be attributed to the temperature‐driven desorption of water molecules from the TiO_2_ surface. This further validates the theory of surface water molecule adsorption and the ion‐proton conduction model.

For the p‐type response to NO_2_ of W‐TiO_2_ at room temperature, NO_2_ reacts with the surface‐adsorbed water molecules in the form of Equation (5). The resulting HNO_3_ and HNO_2_ ionize into NO_2_^−^, NO_3_^−^, and H^+^ in the adsorbed water layer (Figure 8c). Compared with the previous adsorbed H_2_O layers, the amounts of ions after NO_2_ interaction increase, and therefore the surface ionic conductivity of the W‐TiO_2_ enhances. In this way, the resistance of the W‐TiO_2_ decreases as exposed to NO_2_, resulting in the p‐type response. Based on this theory, an increase in humidity in the atmosphere should theoretically enhance the response to NO_2_, which has been confirmed by the aforementioned experimental results in Figure 4d. Additionally, a higher operating temperature will promote the desorption of adsorbed water molecules from the TiO_2_ surface, weakening the charge transfer between the gas and the adsorbed H_2_O layer on the TiO_2_ surface. As a result, the ionic conductivity no longer dominates the conductivity of the W‐TiO_2_. Furthermore, high temperatures provide sufficient activation energy for reactions and facilitate the adsorption of stronger oxidizing oxide anions (O^−^, O^2−^). This allows the traditional space charge model‐based sensing mechanism to regain the dominance. Therefore, at higher temperatures, W‐TiO_2_ should exhibit normal n‐type responses to gases like NO_2_. This hypothesis has been confirmed by demonstrating that the responses of W‐TiO_2_ to all the gases at 400 °C are all n‐type (Figure S6, Supporting Information). All of the above analyses have demonstrated that the anomalous p‐type response to NO_2_ of the W‐TiO_2_ at room temperature could be attributed to H_2_O adsorption induced ion‐proton conduction model.

The normal n‐type response to the reductive NH_3_ of the W‐TiO_2_ could also be explained based on this ion‐proton conduction model. When NH_3_ contacts the water layer on the W‐TiO_2_ surface, the NH_3_ can dissolve in the water layer and dissociate into NH_4_^+^ and OH^−^ (Equation (6), Figure 8c). In this case, the ionic conductivity is enhanced, and the resistance of W‐TiO_2_ decreases, resulting in an n‐type response.

The mechanism for the p‐type response of the W‐TiO_2_ to reducing volatile organic compounds (VOCs), including isopropanol, ethanol, acetone, and formaldehyde, is illustrated as follows. These gases cannot ionize in the adsorbed water layer as organic molecules. The molecule with a greater proton affinity (PA) will retain the protons from the molecule with a smaller PA.^[^ ^69^ ^]^ The VOCs used in this study all have a greater PA than H_2_O does, as displayed in Table S2 (Supporting Information; obtained from the NIST webbook database). When the W‐TiO_2_ sensor is exposed to these VOCs, using ethanol as an example, the protons will be retained by the ethanol molecules, leading to the formation of C_2_H_5_OH_2_^+^ · (H_2_O)_2_ (Equation (7), Figure 8c). This new ion C_2_H_5_OH_2_^+^ · (H_2_O)_2_ contains a lower ion diffusion coefficient and a larger ion radius than the previous H_3_O^+^ does, thereby reduces the ionic conductivity of the H_2_O layer and increases the resistance of W‐TiO_2_, causing the p‐type response.

Overall, when the W‐TiO_2_ is exposed to NO_2_ and NH_3_, the sensor resistance decreases due to the increased ions in the surface adsorbed H_2_O layer, exhibiting an anomalous p‐type response and a normal n‐type response, respectively; when the W‐TiO_2_ is exposed to isopropanol, ethanol, acetone, and formaldehyde vapors, the sensor resistance increases due to the decreased ion diffusion coefficient, displaying an anomalous p‐type response.

(5)

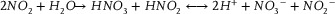



(6)

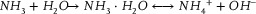



(7)

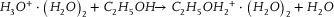




**Figure 9:**
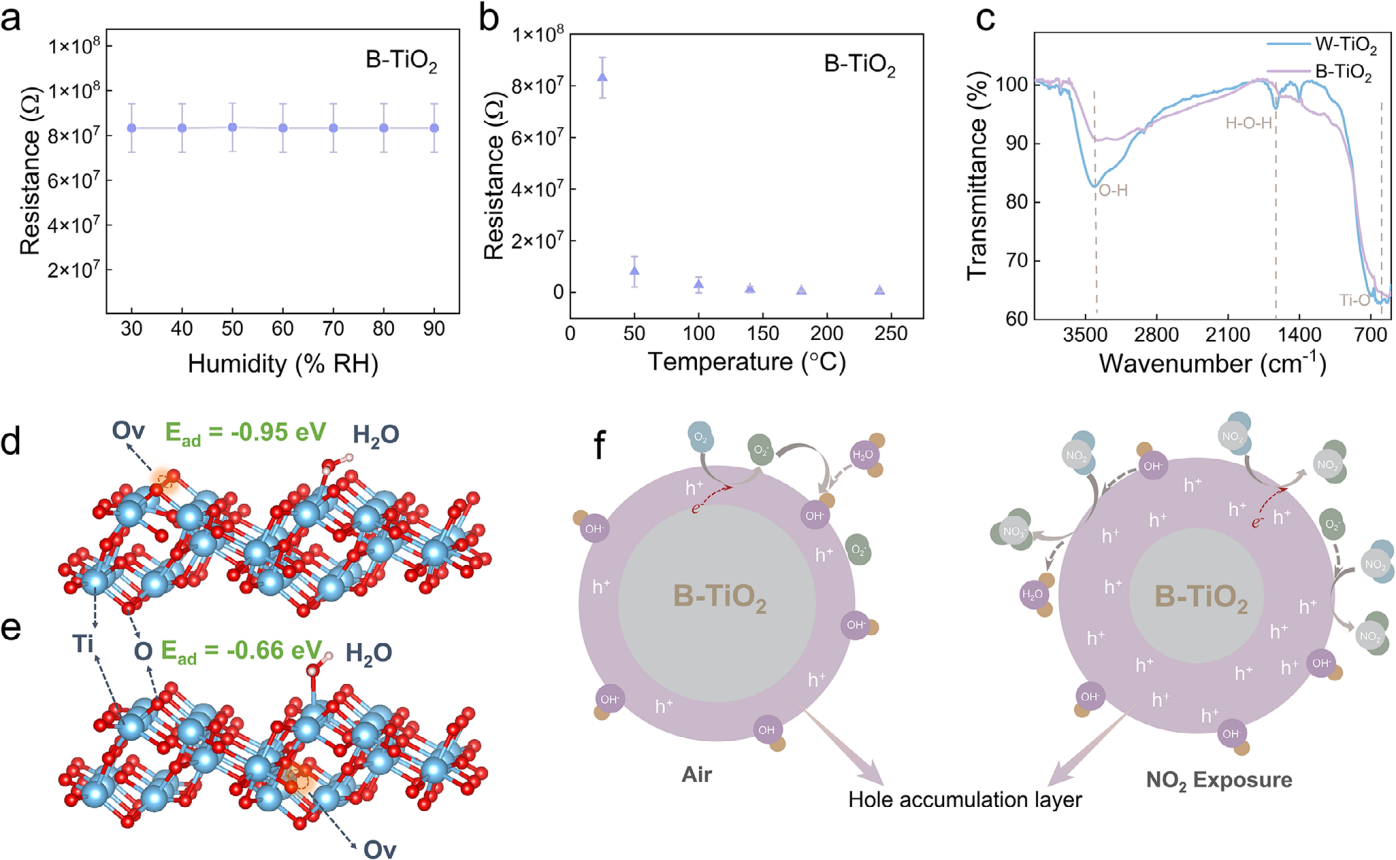
Resistance of B‐TiO_2_ as a function of a) humidity and b) temperature. c) FTIR spectra of W‐TiO_2_ and B‐TiO_2_. DFT models of H_2_O adsorption on TiO_2_ with d) surface O*v* and e) bulk O*v*. f) Schematic diagram of the gas‐sensing mechanism of B‐TiO_2_.

Unlike the W‐TiO_2_, the resistance and the NO_2_ response of the B‐TiO_2_ are not affected by humidity as shown in **Figure** **9**a. To further confirm this, a dynamic cycling measurement of the conductivity of B‐TiO_2_ as the humidity changes back and forth for several times was conducted. The result is shown in Figure S7 (Supporting Information), the resistance shows no obvious fluctuations as the humidity changes. This humidity‐resistant conductivity is consistent with the humidity‐resistant response to NO_2_ mentioned above in Figure 4e and Figure S2 (Supporting Information). Besides, the resistance of the B‐TiO_2_ decreases as the temperature increases in the range from room temperature to 300 °C (Figure 9b), which is also different with the W‐TiO_2_. This increased conductivity caused by the elevated temperature is consistent with the conventional semiconductor theory, which comes from the intrinsic excitation of carriers. These differences suggest that it is not the ion‐proton conduction of the adsorbed H_2_O layer dominating the conductivity of the B‐TiO_2_ as that of the W‐TiO_2_. The form of H_2_O adsorption on the surface of B‐TiO_2_ should be different with that on the W‐TiO_2_, leading to different gas‐sensing mechanisms. To confirm this, Fourier transform infrared spectroscopy (FTIR) measurements were carried out on W‐TiO_2_ and B‐TiO_2_. The results (Figure 9c) show that W‐TiO_2_ exhibits a bending vibration peak of water molecules at 1630 cm^−1^, which confirms the adsorption of H_2_O layers. The B‐TiO_2_ does not exhibit a peak at 1630 cm^−1^, confirming that the surface of the B‐TiO_2_ does not have a H_2_O layer like the W‐TiO_2_. Only the peak at 3400 cm^−1^ is observed in the FTIR spectrum of the B‐TiO_2_, which indicates that the adsorbed H_2_O is mainly in the form of a chemisorbed OH layer. This difference might be caused by the different H_2_O adsorption capabilities between the W‐TiO_2_ and B‐TiO_2_. The adsorption form of water molecules on the TiO_2_ surface varies as the adsorbed H_2_O amount changes. As the adsorbed H_2_O increases, the water molecules are chemically adsorbed on the surface of MOX, forming OH groups first, followed by an additional physically adsorbed water layer,^[^ ^71^ ^]^ which is shown in Figure S8 (Supporting Information). In this way, the dominant conductivity of the MOX changes from electronic conduction to proton‐ion conduction. DFT calculations are conducted to compare the H_2_O adsorption on the W‐TiO_2_ and B‐TiO_2_. As shown by Figure 9d,e, the adsorption energy (*E*_ad_) of H_2_O on W‐TiO_2_ (−0.95 eV) is more negative than that on B‐TiO_2_ (−0.66 eV). This indicates that H_2_O adsorption is suppressed on the B‐TiO_2_ than that on the W‐TiO_2_. This suppressed H_2_O adsorption might contribute to the remarkably enhanced humidity resistance in conductivity and NO_2_ detection performance. This suppressed H_2_O adsorption capability of B‐TiO_2_ than W‐TiO_2_ might be attributed to the less surface oxygen vacancies on B‐TiO_2_, as demonstrated by the XPS and PL results shown in Figure 3b,d. Because the surface O*v* acts as a preferential sites that promote water adsorption.^[^ ^72^ ^]^ Besides, it is reported that p‐type semiconductors exhibit better moisture resistance than n‐type semiconductors.^[^ ^60^, ^73^ ^]^ This may also be a reason for the suppressed water adsorption capability of B‐TiO_2_, because B‐TiO_2_ and W‐TiO_2_ are p‐type and n‐type, respectively. Therefore, the water molecules are chemically adsorbed on the surface of B‐TiO_2_, forming OH groups, while an additional physically adsorbed water molecule layer is formed on the surface of W‐TiO_2_.

This increased OH groups on B‐TiO_2_ are consistent with the aforementioned XPS results (Figure 3b) and might be attributed to the increased adsorption of O_2_^−^. At room temperature, oxygen adsorbs on the sensor surface to form O_2_^−^ (Equation (8)). Then the O_2_^−^ dissociates H_2_O into OH groups (Equation (9)). The surface OH groups will facilitate the NO_2_ adsorption.^[^ ^74^ ^]^ When B‐TiO_2_ is exposed to NO_2_, NO_2_ can easily react with OH on the surface as in Equation (10),^[^ ^63^, ^74^ ^]^ which consumes the electrons in the B‐TiO_2_, causing a relative increase in the hole concentration and thus an increase in conductivity of the B‐TiO_2_ (Figure 9f). Besides, as an oxidizing gas with electron‐trapping properties, NO_2_ usually acts as an electron acceptor, trapping electrons from the conduction band of TiO_2_, and the reaction of Equation (11) occurs.^[^ ^75^, ^76^ ^]^ In addition, NO_2_ also interacts with surface O_2_^−^ to form NO_2_^−^, a process that also consumes electrons from TiO_2_ (Equation (12)).^[^ ^75^, ^76^ ^]^ All of the above processes broaden the hole accumulation layer and significantly increase the electrical conductivity of B‐TiO_2_, as shown in Figure 9f. Upon re‐exposure to air, NO_2_^−^ and NO_3_^−^ dissociate into NO_2_ and release electrons to the CB of TiO_2_, and the resistance recovers. In contrast, a water layer exists on the W‐TiO_2_ surface, which has a blocking effect for NO_2_ to undergo the interactions described above. Therefore, the response of B‐TiO_2_ to NO_2_ is significantly higher than that of W‐TiO_2_.

The OH group, as a polar functional group, has a hydrogen‐bonding capability, allowing it to form a hydrogen bond with the nitrogen and oxygen atoms in the gas molecules. This allows the adsorption of NH_3_, isopropanol, ethanol, formaldehyde, and acetone gases on B‐TiO_2_ in the form of Equation (13), where R represents the above gases. However, the formation of hydrogen bonds only changes the distribution of electrons between the OH groups and the adsorbed gas molecules, hardly affecting the carriers in the B‐TiO_2_.^[^ ^77^, ^78^ ^]^ The adsorbed gas molecules can also undergo redox reactions with the surface O_2_^−^ while this reaction path is limited due to the required activation energy and decreased O_2_^−^ caused by the reaction (9). As a result, B‐TiO_2_ shows suppressed responses to these gases.

(8)

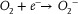



(9)

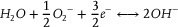



(10)

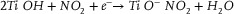



(11)

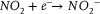



(12)

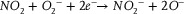



(13)

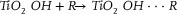




## 3 Conclusion

In summary, a room‐temperature‐operated NO_2_ sensor with ultrahigh selectivity and humidity resistance is developed using black TiO_2_ (named B‐TiO_2_). The B‐TiO_2_ is obtained through defect engineering by annealing TiO_2_ in vacuum, which contains much more bulk oxygen vacancies than the commonly white TiO_2_ (named W‐TiO_2_) does. Compared to W‐TiO_2_, B‐TiO_2_ enhances the response to NO_2_ by more than 10 times and suppresses the response to several interference gases simultaneously, therefore greatly improves the selectivity to NO_2_. Besides, the B‐TiO_2_‐based sensor shows enhanced humidity immunity than the W‐TiO_2_ does by suppressing the H_2_O adsorption. A portable wireless NO_2_ sensor system is developed herein which achieves a specific detection of NO_2_ in a mixture of 7 gases and successfully detects the environmental differences between the traffic intersection and the indoor. Besides, the W‐TiO_2_ and B‐TiO_2_ exhibit different gas response types, and the response types are dependent on the gas species. The mechanisms for the different response types and the significantly enhanced sensing performance of B‐TiO_2_ are revealed using material characterization, response comparison, gas–solid interactions study, and DFT analysis. It is the different surface adsorption models of H_2_O that determine the different sensing behaviors of B‐TiO_2_ and W‐TiO_2_. The H_2_O layers adsorbed on the surface of the W‐TiO_2_ allow ionic conductivity to dominate the conductivity and block the interaction between NO_2_ and W‐TiO_2_, while the OH groups on the B‐TiO_2_ surface enhance the response and selectivity to NO_2_. This work has established a novel understanding of the gas sensing mechanism and provided a new effective way to improve the sensing performance of MOX‐based NO_2_ sensors at room temperature.

## 4 Experimental Section

### Synthesis of TiO_2_ Burr‐Like Nanorods and the Corresponding Sensors

TiO_2_ burr‐like nanorods were synthesized by the liquid precipitation method at room temperature. The specific steps are shown in Figure 1a. 0.1 mol L^−1^ (NH_4_)_2_TiF_6_ (Anhui Senrise Technology Co., Ltd) and 0.3 mol L^−1^ H_3_BO_3_ (Anhui Senrise Technology Co., Ltd.) were mixed uniformly first. Then the ZnO nanowires (Jiangsu XFNANO Materials Tech Co, Ltd.) were immersed into the above solution at room temperature for 50 h. White products appeared at the bottom of the solution. The product was separated from the reaction solution by centrifugation and washed with deionized (DI) water to finally obtain the TiO_2_ burr‐like nanorods. The detailed descriptions can be found in the previous publication.^[^ ^79^ ^]^

The TiO_2_ burr‐like nanorods were mixed with DI water to form a paste, 3 µL paste was uniformly drop‐coated onto a ceramic substrate (provided by Beijing Ailite Technology Co., Ltd., which contains a pair of electrodes on each side, one for resistance measurement and the other for heating) to form sensing film (Figure S9, Supporting Information). Then the substrate was heated for 5 min at 70 °C on a hot plate to remove the water, followed by annealing at 500 °C for 4 h: the white TiO_2_ was obtained by annealing in air and the black TiO_2_ was obtained by annealing in vacuum. Finally, the annealed ceramic substrates were welded into a six‐pin base (Figure S9, Supporting Information). By now, the fabrication of gas sensors was completed. The sensors based on white TiO_2_ and black TiO_2_ were labeled as W‐TiO_2_ and B‐TiO_2_, respectively.

### Sensing Measurements

The gas sensing performance of the sensor was measured using an intelligent gas sensing analysis system (CGS‐8, Beijing Ailite Technology Co., Ltd.), as shown in Figure S10 (Supporting Information). The static gas distribution method was used. For NO_2_, CO_2_, and H_2_, the atmospheres of various concentrations were controlled by injecting different volumes of gas into the chamber. For NH_3_ and the VOCs, the corresponding amount liquid was dropped into the chamber, followed by volatilizing, and the gas concentration was derived by the formula of $c=\frac{22.4\times \rho \times d\times V_{1}}{M\times V_{2}}$, where *V*_2_ is the volume of the measurement chamber, *V*_1_, *M*, ρ, and *d* are the volume, relative molecular mass, density, and purity of the liquid, respectively. During the recovery, the chamber was opened and exposed to air. The response of the sensor was defined as follows: when *R*_a_ > *R*_g_, $response=\frac{R_{a}-R_{g}}{R_{g}}\times 100\%$; when *R*_a_ < *R*_g_, $response=\frac{R_{a}-R_{g}}{R_{a}}\times 100\%$ (*R*_a_ and *R*_g_ represent the sensor resistance before and after gas exposure, respectively). Response/recovery time was defined as the time taken by the gas sensor to reach 90% of the total resistance change in case of adsorption and desorption respectively. All the measurements were conducted at room temperature (25 °C).

### Characterizations

XRD patterns were recorded using an X‐ray diffractometer (SmartLab, Rigaku). The morphology and the elemental species were analyzed using FESEM (GeminiSEM 300, ZEISS) and EDS (Max100, Oxford). The elemental content and atomic valence were analyzed using XPS (Escalab Xi+, Thermo Fisher). The UV–vis spectra were obtained using a UV–vis near‐infrared spectrophotometer (Lambda 1050+, PerkinElmer). Defects were characterized using an EPR spectrometer (EMXnano, Bruker) and a fluorescence spectrophotometer (FLS1000, Edinburgh). FTIR spectra were conducted utilizing an FTIR spectrometer (Nicolet iS50, Thermo Fisher Scientific).

### DFT Calculations

The Vienna *ab* initio simulation package was utilized for the following computational calculations. Typically, the generalized gradient approximation with the Perdew–Burke–Ernzerhof functional was used to reveal the exchange and correlation effects. Based on the anatase TiO_2_ structure, the (101) surface containing 36 Ti atoms and 72 O atoms was chosen as a typical calculation model. The vacuum gap was set as 15 Å. The convergence threshold for total energy converged within 10^−5^ eV atom^−1^ and the average force was 0.05 eV Å^−1^. For the grid integration, a cutoff energy of 450 eV was applied and projector augmented wave PAW potentials were selected for describing ion cores. The Brillouin‐zone was sampled by the gamma‐centered Monkhorst–Pack method with a 2 × 2 × 1 k‐point mesh for geometric optimizations.

The adsorption energy (*E_ad_*) was calculated as follows:

(14) $$\mathrm{Ead}=E\left(*\mathrm{ads}\right)-E\left(*\right)-E\left(\mathrm{ads}\right)$$

where *E*(*ads), *E*(*), and *E*(ads) are the total energy of samples with adsorbates on the surface, the energy of the pristine samples surface, and adsorbates, respectively.

## Conflict of Interest

The authors declare no conflict of interest.

## Supporting information



Supporting Information

Supplemental Video 1

## Data Availability

The data that support the findings of this study are available from the corresponding author upon reasonable request.
