# Supplementary material for: An Ultra‐Selective and Humidity‐Resistant Room‐Temperature‐Operated NO2 Sensor Based on Black TiO2
Source: Adv Sci (Weinh). 2025 Aug 11;12(41):e09293. doi: 10.1002/advs.202509293 (PMC12591125; doi:10.1002/advs.202509293)
Supplement: Supplementary file 1 — Supporting Information [file ADVS-12-e09293-s001.docx]

Supporting Information

An ultra-selective and humidity-resistant room-temperature-operated NO_2_ sensor based on black TiO_2_

Xuelan Cheng, Yizheng Liu, Wei Zhong, Shuai Li, Yan Li, Zixi Zhao, Chunlei Zhang, Jidong Shi, Hui Liu, Zonglong Zhu*, Fang Xu*


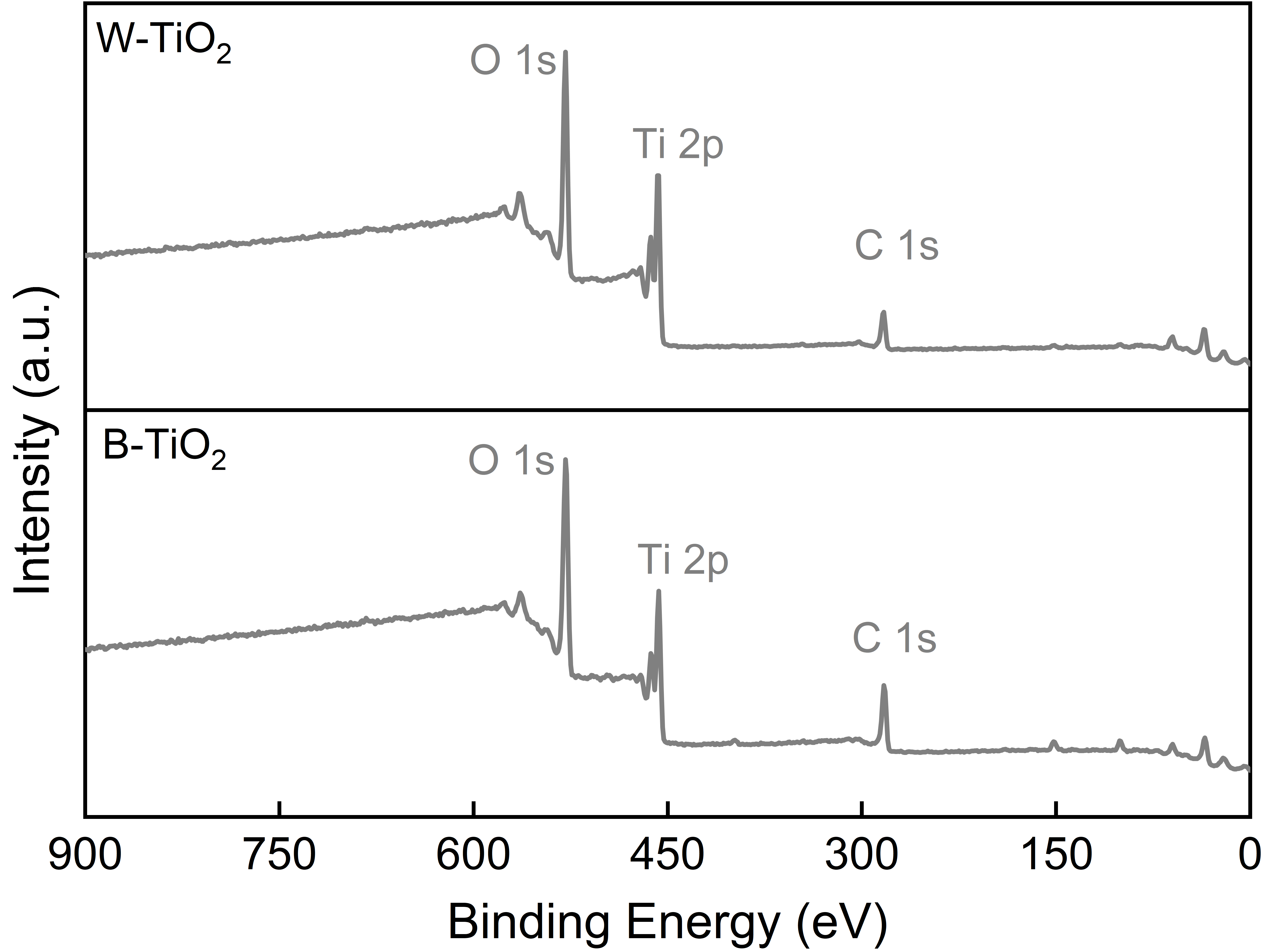


**Figure S1** XPS survey spectra of W-TiO_2_ and B-TiO_2_.


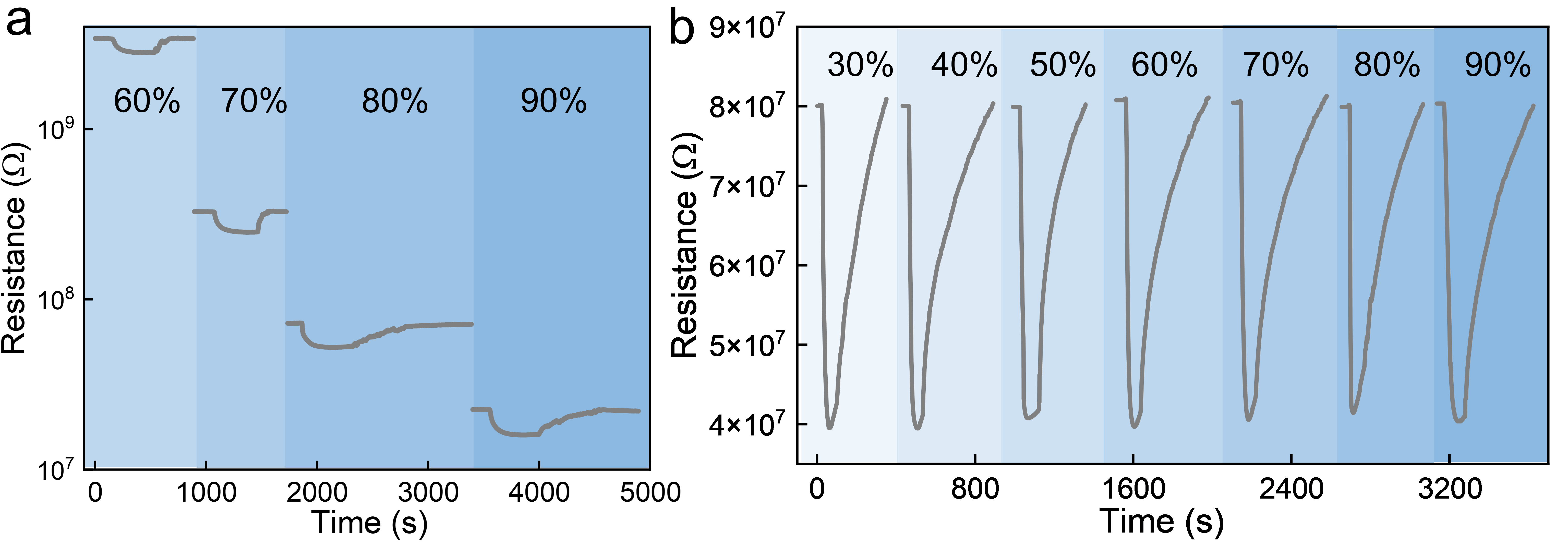


**Figure S2**. a) Dynamic sensing curve of W-TiO_2_ to 10 ppm NO_2_ at 60% to 90% RH. (b) Dynamic sensing curve of B-TiO_2_ to 500 ppb NO_2_ at 30% to 90% RH.





**Figure S3**. Real time sensing curve of B-TiO_2_ to 500 ppb NO_2_ under 50 cycles continuous measurements.





**Figure S4**. Response types to 100 ppm ethanol, isopropanol, acetone, formaldehyde, 50 ppm NH_3_, and 3 ppm NO_2_ of a) W-TiO_2_, b) B-TiO_2_ at room temperature.





**Figure S5**. Dynamic resistance curve of W-TiO_2_ under cyclic humidity variations.


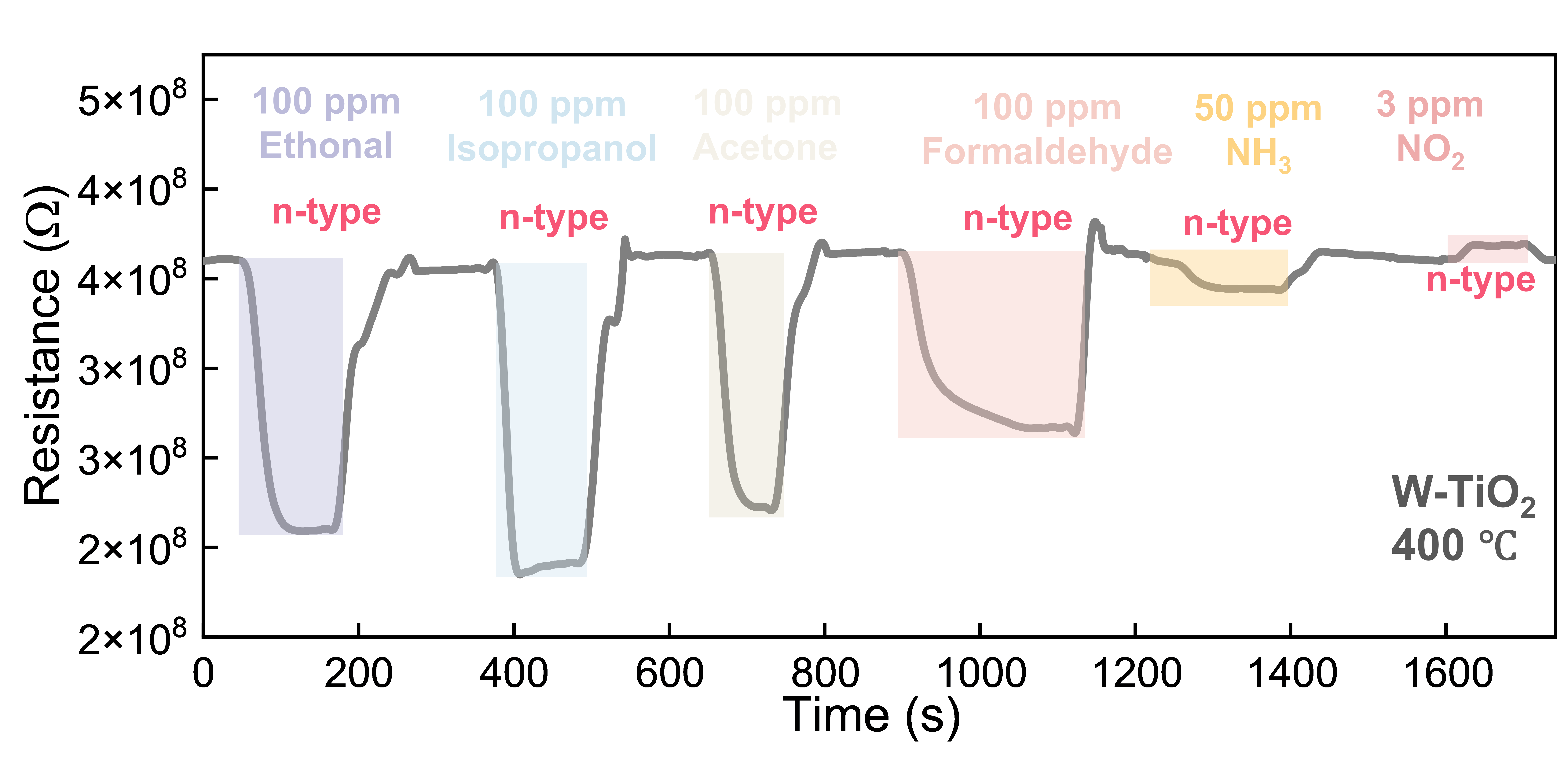


**Figure S6**. Response types to 100 ppm ethanol, isopropanol, acetone, formaldehyde, 50 ppm NH_3_, and 3 ppm NO_2_ of W-TiO_2_ at 400 °C.





**Figure S7**. Dynamic resistance curve of B-TiO_2_ under cyclic humidity variations.


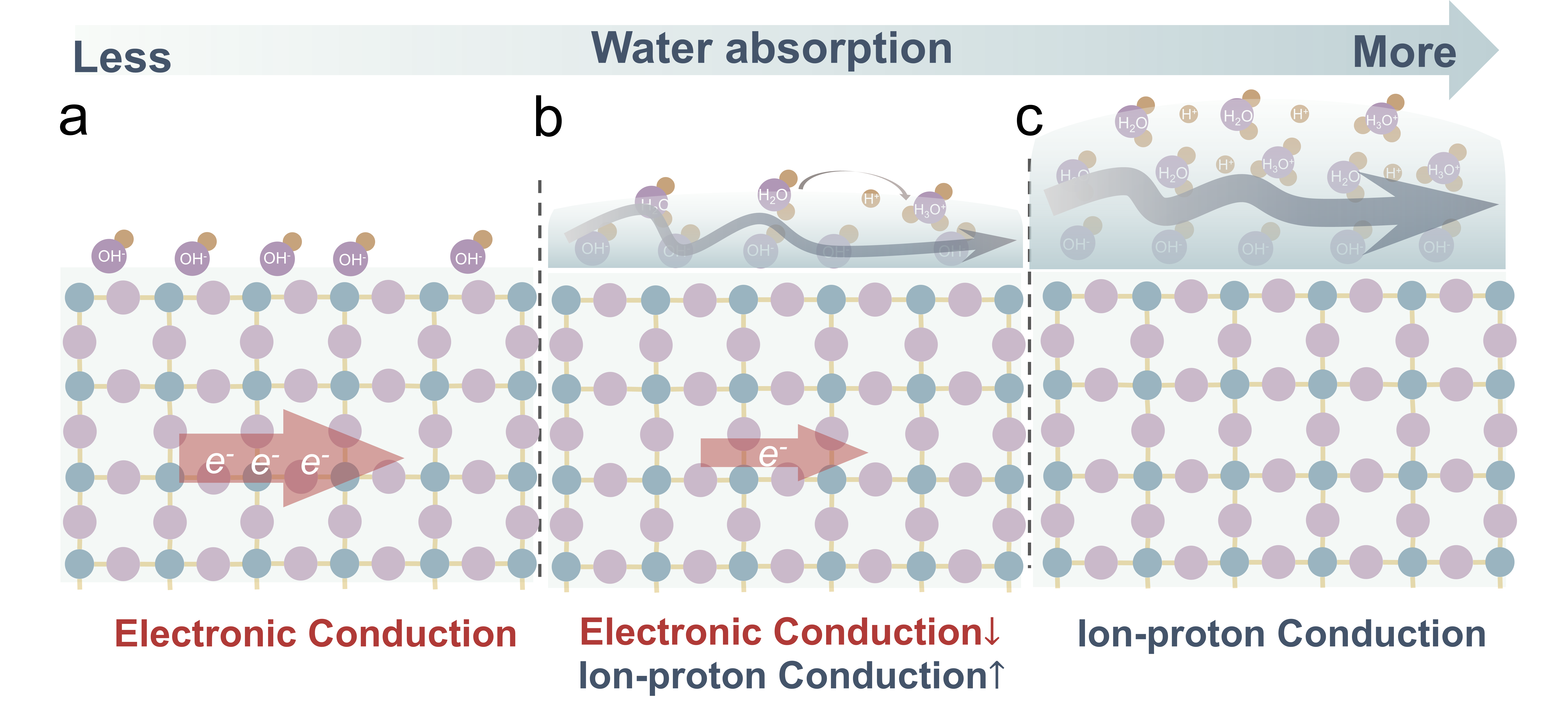


**Figure S8** Schematics of water absorption forms on MOX and corresponding dominant conductivity.


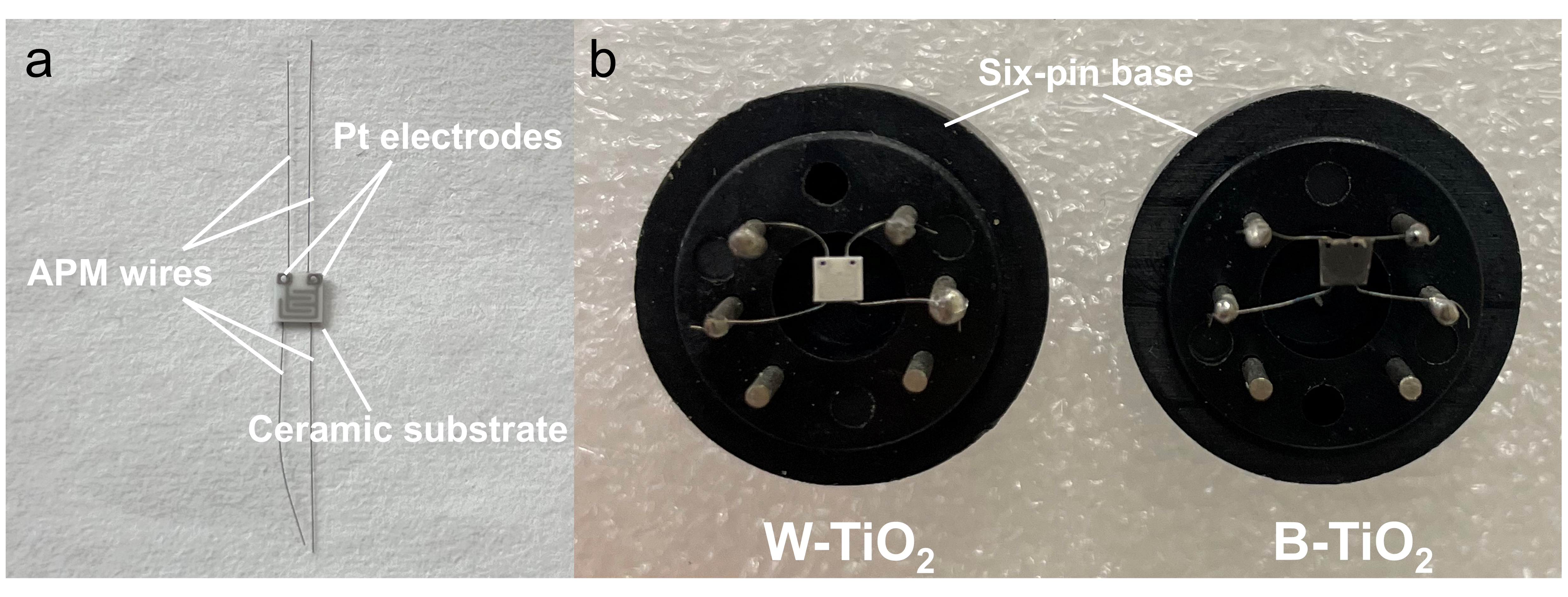


**Figure S9** Actual appearance of a) the blank ceramic substrate, b) the sensors labeled as W-TiO_2_ and B-TiO_2_.


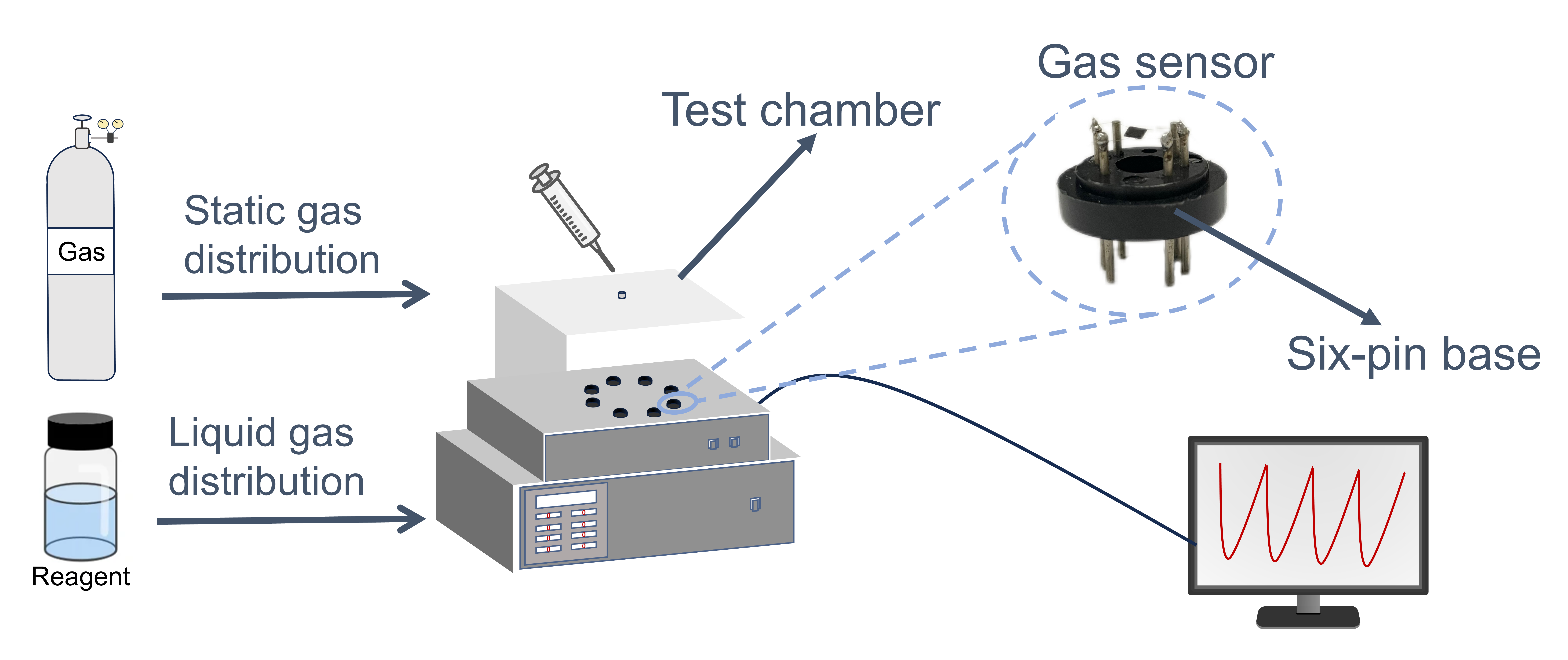


**Figure S10** Schematic diagram of the sensing performance measurement.

**Table S1** Hall measurement results of W-TiO_2_ and B-TiO_2_ thin films.

|  | Temperature | Sample Thickness | Carrier Type | Hall voltage |
| --- | --- | --- | --- | --- |
| W-TiO_2_ | 300 K | 100 nm | n-type | -1440 $\text{μV}$ |
| B-TiO_2_ | 300 K | 100 nm | p-type | 460 $\text{μV}$ |

**Table S2** Proton affinity of water and the measured VOCs.

|  | Water | Ethanol | Isopropanol | Acetone | formaldehyde |
| --- | --- | --- | --- | --- | --- |
| PA (Kcal/mol) | 165.2 | 185.6 | 189.5 | 194.1 | 170.4 |
